# Massive horizontal gene transfer, strictly vertical inheritance and ancient duplications differentially shape the evolution of *Bacillus cereus* enterotoxin operons *hbl, cytK* and *nhe*

**DOI:** 10.1186/s12862-015-0529-4

**Published:** 2015-11-10

**Authors:** Maria-Elisabeth Böhm, Christopher Huptas, Viktoria Magdalena Krey, Siegfried Scherer

**Affiliations:** Lehrstuhl für Mikrobielle Ökologie, Zentralinstitut für Ernährungs-und Lebensmittelforschung (ZIEL), Technische Universität München, Weihenstephaner Berg 3, D-85350 Freising, Germany; Current address: BCA-clinic Betriebs GmbH & Co. KG, Morellstr. 33, D-86159 Augsburg, Germany

**Keywords:** *Bacillus cereus* sensu lato, Average nucleotide identity, Enterotoxin, *nhe*, *hbl*, *cytK*, Horizontal gene transfer, gene duplication, Functional constraints of evolution

## Abstract

**Background:**

*Bacillus cereus* sensu lato comprises eight closely related species including the human pathogens *Bacillus anthracis* and *Bacillus cereus*. Within *B. cereus* sensu lato, chromosomally and plasmid-encoded toxins exist. While plasmid-mediated horizontal gene transfer of the emetic toxin, anthrax and insecticidal toxins is known, evolution of enterotoxin genes within the group has not been studied.

**Results:**

We report draft genome assemblies of 25 strains, a phylogenetic network of 142 strains based on ANI derived from genome sequences and a phylogeny based on whole-genome SNP analysis. The data clearly support subdivision of *B. cereus* sensu lato into seven phylogenetic groups. While group I, V and VII represent *B. pseudomycoides*, *B. toyonensis* and *B. cytotoxicus*, which are distinguishable at species level (ANI border ≥ 96 %), strains ascribed to the other five species do not match phylogenic groups. The chromosomal enterotoxin operons *nheABC* and *hblCDAB* are abundant within *B. cereus* both isolated from infections and from the environment. While the duplicated *hbl* variant *hbl*_*a*_ is present in 22 % of all strains investigated, duplication of *nheABC* is extremely rare (0.02 %) and appears to be phylogenetically unstable. Distribution of toxin genes was matched to a master tree based on seven concatenated housekeeping genes, which depicts species relationships in *B. cereus* sensu lato as accurately as whole-genome comparisons. Comparison to the phylogeny of enterotoxin genes uncovered ample evidence for horizontal transfer of *hbl, cytK* and *plcR,* as well as frequent deletion of both toxins and duplication of *hbl*. No evidence for *nhe* deletion was found and stable horizontal transfer of *nhe* is rare. Therefore, evolution of *B. cereus* enterotoxin operons is shaped unexpectedly different for yet unknown reasons.

**Conclusions:**

Frequent exchange of the pathogenicity factors *hbl, cytK* and *plcR* in *B. cereus* sensu lato appears to be an important mechanism of *B. cereus* virulence evolution, including so-called probiotic or non-pathogenic species, which might have consequences for risk assessment procedures. In contrast, exclusively vertical inheritance of *nhe* was observed, and since *nhe*-negative strains appear to be extremely rare, we suggest that fitness loss may be associated with deletion or horizontal transfer of the *nhe* operon.

**Electronic supplementary material:**

The online version of this article (doi:10.1186/s12862-015-0529-4) contains supplementary material, which is available to authorized users.

## Background

*Bacillus cereus* sensu lato comprises eight species of gram-positive, endospore-forming bacteria with greatly varying pathogenic potential. *B. cereus* sensu stricto, which was discovered in 1887 as a bacterium occurring ubiquitously in nature [1], contains probiotic as well as pathogenic strains. The latter are mostly associated with food-borne illness characterized by diarrhea or vomiting, but occasionally *B. cereus* is responsible for severe infections, e.g. endophthalmitis or meningitis [2]. The emetic type of *B. cereus* food poisoning is caused by ingestion of the small, cyclic and heat-stable toxin cereulide [3]. The three most important and well-known enterotoxins are the non-hemolytic enterotoxin (Nhe), hemolysin BL (Hbl) and cytotoxin K (CytK). *B. thuringiensis* produces insecticidal parasporal protein crystals of Cry (crystal) and/or Cyt (cytolytic) proteins that are mostly encoded on plasmids. Occasionally *B. thuringiensis* have been found to cause human infections very similar to *B. cereus* [4, 5]. *B. anthracis* is the best known human and animal pathogen of the *B. cereus* group and was demonstrated to be the causative agent of anthrax by Robert Koch in 1876 [6]. The anthrax-associated plasmids pXO1 (encoding anthrax toxin genes *pag, lef* and *cya*) and pXO2 (encoding the poly-γ-D-glutamic acid capsule genes *cap*) have been found in a few *B. cereus* strains such as *B. cereus* G9241 and *B. cereus* biovar *anthracis* CA [7, 8] with a similar pathogenic potential as *B. anthracis. B. weihenstephanensis* is psychrotolerant and able to grow below 7 °C [9]. Occasionally, this species houses the emetic toxin cereulide [10, 11]. The psychrotolerant *B. mycoides* is closely related to the other *B. cereus* sensu lato species (16S rRNA sequences showed > 99 % identity [12]), but it can easily be distinguished by its rhizoidal colonial growth [13]. To our knowledge, no infections by *B. mycoides* have been reported, but it carries both *nhe* and *hbl* and its cytotoxicity was shown [14]. Within *B. mycoides* a group of bacteria with a clearly distinguishable fatty acid profile was recognized and described as *B. pseudomycoides* [15]. In 1998, a highly enterotoxic and rare variant of cytotoxin K, CytK-1, was discovered in *B. cereus* NVH 391–98, a strain responsible for severe food poisoning. This strain was published in 2013 as the type strain of the new species *B. cytotoxicus* on the basis of presence of the *cytK-1* gene, its thermotolerance (growth at up to 50 °C), a distinctive fatty acid profile, DNA-DNA hybridization and multilocus sequence typing (MLST) [16]. The eighth member of *B. cereus* sensu lato was isolated in Japan in 1966 but has been described as a separate species *B. toyonensis* only recently [17]. Interestingly, it is a commercially available probiotic (TOYOCERIN®). *B. toyonensis* was distinguished from other *B. cereus* sensu lato type species by pairwise calculations of the average nucleotide identity (ANI).

While the trend in prokaryotic species distinction is moving towards comparison of entire genomes [18, 19], differentiation of the closely related *B. cereus* species is still based on the presence or absence of phenotypic characters. It has been reported that species affiliation of *B. cereus* group strains often does not match the phylogenetic relatedness [12, 20, 21]. One reason for such discrepancies may be the exchange of virulence plasmids between species. Plasmids pXO1 and pXO2 encoding the anthrax toxin complex and the poly-γ-D-glutamic acid capsule are found not only in *B. anthracis*, but also in some *B. cereus* strains [7, 22, 23]. The *B. cereus* cereulide synthetase gene cluster is also located on a large pXO1-like plasmid [3] and is not restricted to a single lineage within *B. cereus* sensu lato [11, 24]. Some of the *B. thuringiensis* insecticidal *cry* genes are encoded on plasmids [25] and can spread via horizontal gene transfer (HGT) among *B. cereus* sensu lato. Therefore, the transfer of a single plasmid from one species to another species may result in a change of species affiliation.

Several studies have addressed the general possibility of HGT among *B. cereus* sensu lato, obtaining controversial results. *B. cereus* and *B. thuringiensis* isolates have been studied by multilocus enzyme electrophoresis (MLEE) resulting in a high variety of closely related electrophoretic types, evidence of extensive recombination between species and a low degree of clonality [26]. MLST analyses revealed that *B. anthracis* is a homogeneous and clonal cluster within *B. cereus* sensu lato, while *B. cereus* and *B. thuringiensis* are of higher diversity [27]. Similar studies have been conducted with a different set of housekeeping genes for MLST, amplified fragment length polymorphism (AFLP) and genomic comparisons, concluding that HGT involves chromosomal genes and is probably mediated by transposable elements [26, 28, 29]. Other studies conclude from MLST, AFLP, MLEE and genomic data that chromosomal recombination events are generally rare, but appear more often among *B. cereus/B. thuringiensis*, while only plasmids are transmitted by HGT [30–32]. HGT among distantly related bacteria can be inferred by several approaches [33], but these are difficult to apply when bacteria as closely related as *B. cereus* sensu lato are investigated [34]. Conflicting results are therefore not surprising.

While horizontal spread of virulence plasmids is not unusual, little is known about the lateral transfer of chromosomal *B. cereus* virulence factors [27, 29]. Therefore, this work aims to systematically evaluate the prevalence and distribution of enterotoxin genes within *B. cereus* sensu lato, with a focus on horizontal versus vertical gene transfer as well as toxin gene duplication and loss. As an indispensable prerequisite for this task we inferred the phylogeny of *B. cereus* sensu lato strains based on 142 genomes (25 of which we sequenced *de novo* in this work) as well as stable MLSA (multilocus sequence analysis) phylogeny constructed from seven housekeeping genes of the *B. cereus* core genome.

## Results and Discussion

### *De novo* sequencing of 25 *B. cereus* sensu lato strains

In this study whole genomes of 25 *B. cereus* sensu lato strains with different enterotoxic potential (for details see [35]) have been sequenced. 21 of them are known members of *B. cereus* sensu stricto that were either isolated from food or associated with food poisoning cases. *B. cereus* #17 (#236) has been isolated from mouse gut (T. Clavel, personal communication) and *B. cereus* IP5832 (#237) is a commercially available probiotic strain (Bactisubtil®) [36]. Additionally, *B. mycoides* WSBC 10969 (#283), and *B. cytotoxicus* CVUAS 2833 (#249) [37] were added, because only very few genomes of these species are publicly available. Detailed sequencing and assembly data are given in Additional file 1: Table S1. Assembly sizes of the newly sequenced strains ranged from 4.1 to 6.8 Mbp (Table 1) and were compared to genome sizes of published *B. cereus* sensu lato strains. It has been discussed controversially whether well-adapted pathogenic bacteria generally contain smaller genomes (due to less variable selection pressure) than environmental isolates [38]. In our study, some *B. cereus* sensu lato genomes seem to support this hypothesis of a reduced genome size in pathogens, such as *B. anthracis* (5.0 – 5.5 Mbp), *B. cytotoxicus* (4.1 Mbp) and some of the enteropathogenic strains we sequenced. These showed smaller genomes than environmental and innocuous strains (*B. thuringiensis* 5.3 – 6.8 Mbp, *B. mycoides* 5.6 – 6.1 Mbp, *B. pseudomycoides* 5.8 Mbp). However, several exceptions like the enterotoxic Nhe reference strain *B. cereus* NVH 0075–95 (6.1 Mbp) support the notion that genome size does not correlate with pathogenicity.

**Table 1:** Genome size and pathogenicity of 25 *de novo* sequenced *B. cereus* sensu lato strains

Strain	Assembly size [bp]	Source	Enterotoxicity
*B. cytotoxicus* CVUAS 2833	4127075	Food poisoning	N.d.
*B. cereus* F4429/71	5284967	Vanilla pudding	High
*B. cereus* HWW 274-2	5290159	Milk powder	Low
*B. cereus* SDA KA 96	5335844	Raw milk	High
*B. cereus* RIVM BC 126	5417487	Human faeces	High
*B. cereus* 7/27/S	5479572	Human faeces	High
*B. cereus* 14294–3 (M6)	5523305	Ice cream	Middle
*B. cereus* MHI 86	5551873	Infant food	Low
*B. cereus* RIVM BC 90	5559670	Human faeces	Low
*B. cereus* F4430/73	5577793	Pea soup	Middle
*B. cereus* F3162/04	5591156	Human faeces	High
*B. cereus* IP5832	5592318	Probiotic	N.d.
*B. cereus* INRA C3	5596453	Carrot	High
*B. cereus* WSBC 10035	5619577	Milk	High
*B. weihenstephanensis* WSBC 10204	5655039	Milk	N.d.
*B. cereus* F3175/03	5733808	Human faeces	Middle
*B. cereus* RIVM BC 964	5815402	Kebab	High
*B. cereus* #17	5852222	Commensal	N.d.
*B. cereus* F528/94	5935300	Food poisoning	Low
*B. cereus* INRA A3	6075647	Starch	Low
*B. mycoides* WSBC 10969	6101972	Raw milk	N.d.
*B. cereus* NVH 0075-95	6112682	Food poisoning	High
*B. cereus* MHI 226	6233017	Milk	Low
*B. cereus* 6/27/S	6771128	Human faeces	Middle
*B. cereus* RIVM BC 934	6840916	Lettuce	Low

Strains are sorted by assembly size. Classification in high/low enterotoxic strains was determined by using a Vero cell assay [35]. N. d.: not determined

After assembly, the new sequences were screened for the presence and location of virulence determinants and housekeeping genes and used for whole-genome comparison as well as multi-locus sequence analysis.

### Whole genome comparison confirms seven phylogenetic groups

**Fig. 1:**
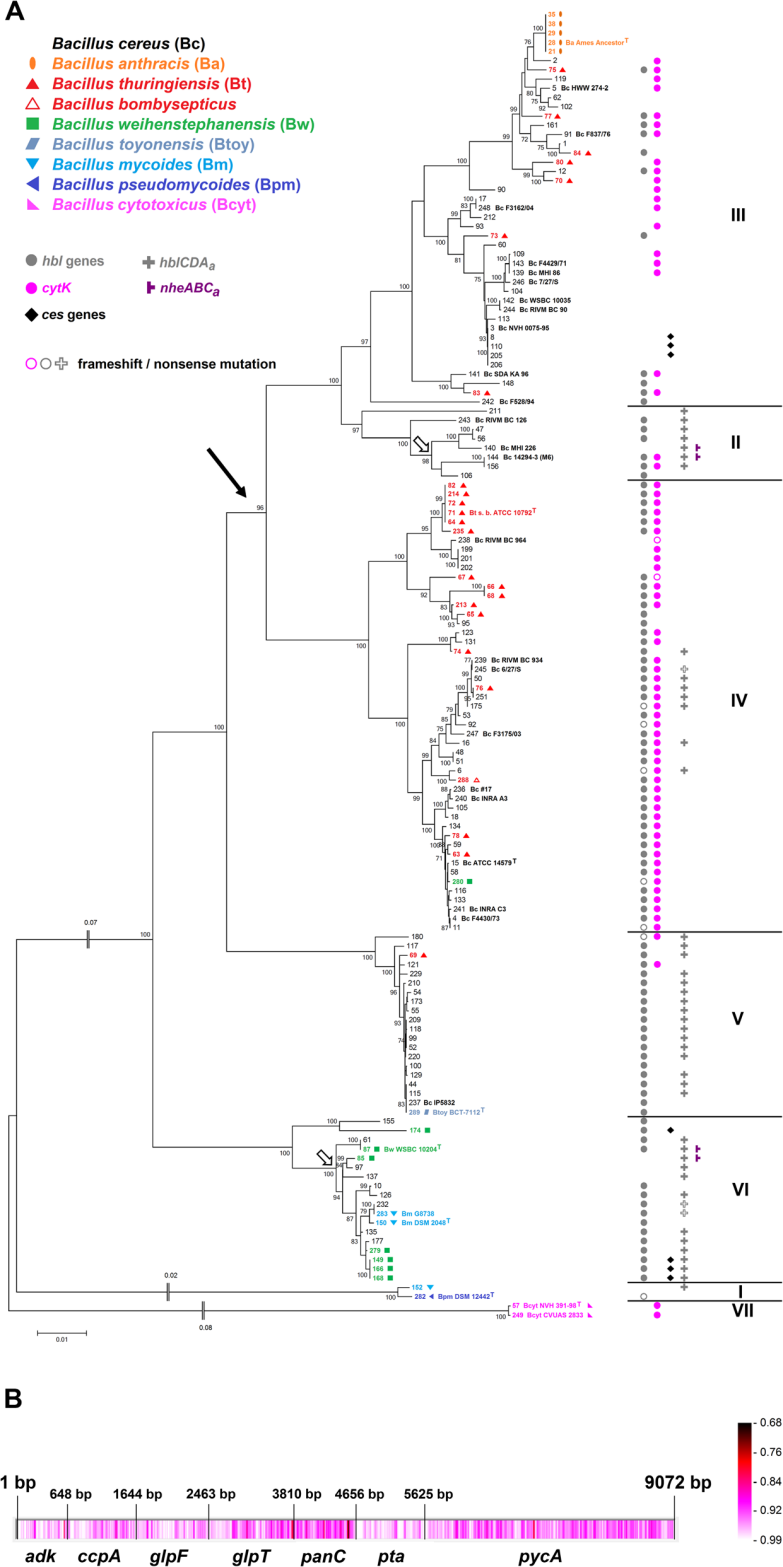
MLSA-based species relationship within *B. cereus* sensu lato. **a**: The phylogenetic tree (Maximum Likelihood Method) was calculated using the concatenated sequence of seven housekeeping genes from 142 *B. cereus* sensu lato strains. Arrow: Suspected first appearance of *cytK-2*. Empty arrows: Suspected origin of *nhe*_*a*_ operons. **b**: Visualization of the sequence homology derived from a multiple sequence alignment calculated with RDP3. Color ranges (identity score: ≤ 1, 1 = identical in all sequences) from identical (white) to highly dissimilar (black)

**Fig. 2:**
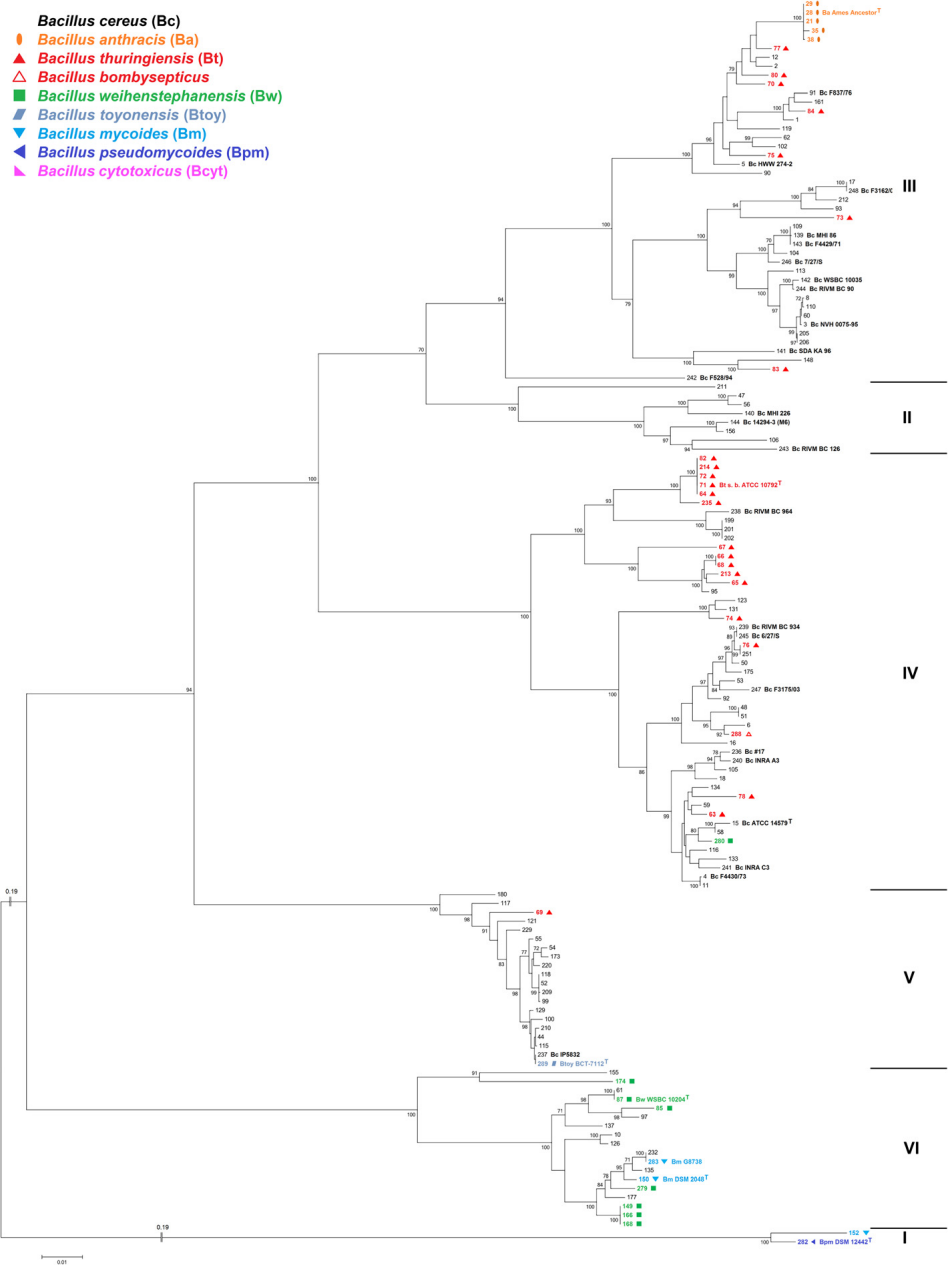
SNP-based species relationship within *B. cereus* sensu lato. The phylogenetic tree (Maximum Likelihood Method) was calculated using the genome-wide core SNP matrix (SNPs that are present in all of the analyzed genomes) of 140 *B. cereus* sensu lato genomes. Phylogenetic cluster VII is too divergent for accurate detection of core SNPs using kSNP3 and had to be excluded

**Fig. 3:**
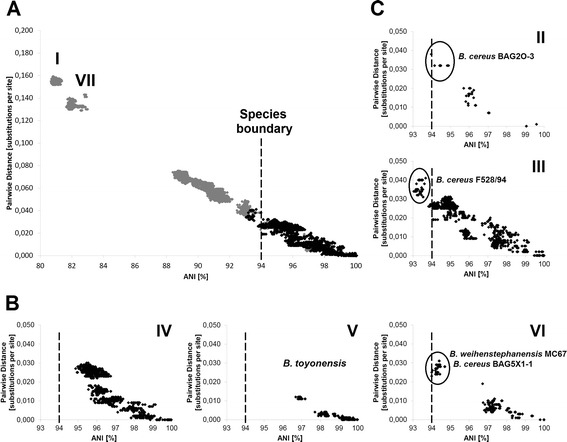
Correlation of pairwise distance of concatenated housekeeping genes with whole genome ANI values. **a**: Correlation of whole-genome ANI and pairwise distance of seven concatenated housekeeping genes of all 142 *B. cereus* sensu lato strains. Intra-cluster values (for clusters see Fig. 1) are depicted in black, all inter-cluster values in grey. Pearson’s correlation of all values is r^2^ = 0.974. **b** and **c**: Intra-cluster comparison of whole-genome ANI and pairwise distance of seven concatenated housekeeping. Cluster affiliation (I – VII) according to species tree (Fig. 1). Strains that are difficult to sort into a distinct genomospecies are named and marked by circles. **b**: Clusters I, IV – VI can be distinguished at an ANI species boundary of ≥ 94 %. Cluster I: ANI 98.3 %, pairwise distance 0.005 substitutions per site. Cluster VII: ANI 99.5 %, pairwise distance 0.001 substitutions per site. **c**: Clusters II and III cannot be discerned on the basis of the comparisons presented: strain *B. cereus* F528/94 (#242) is affiliated to Cluster III by MLSA (Fig. 1) and whole-genome SNP analysis (Fig. 2), but to Cluster II by ANI (Additional file 2: Figure S1)

For taxonomical purposes, and in order to analyze horizontal gene transfer, the construction of a phylogenetic master tree which depicts the assumed “true” phylogenetic relationships of the organisms studied as correctly as possible is mandatory. Towards this end, we constructed an MLSA tree (Fig. 1) based on concatenated sequences of seven housekeeping genes [39] from the *B. cereus* sensu lato core genome. The topologies of trees calculated on the basis of individual housekeeping genes are highly similar to this master tree but partially lack resolution due to different levels of conservation (data not shown). The overall MLSA tree topology was confirmed by an analysis of whole-genome pairwise ANI comparison, visualized by a neighbor network (Additional file 2: Figure S1). Pairwise comparison of ANI versus pairwise distances of the seven concatenated housekeeping genes correlated nicely (Fig. 3a). These results were additionally confirmed by whole-genome SNP-based phylogeny (Fig. 2). All three methods showed highly similar tree topologies and confirmed that the MLSA tree should correctly display the strain phylogeny of the 142 *B. cereus* sensu lato strains included in this study.

Based on a more limited MLST approach, using partial gene sequences only as well as a different set of genes than we applied in our MLSA analysis, Cardazzo *et al.* [28] concluded that reticulate evolution of housekeeping genes should be an important factor of *B. cereus* evolution. Based on our whole genome and MLSA analysis we cannot exclude limited reticulate evolution within the seven housekeeping genes used in our study but we suggest that, apparently, this does not mask the strain phylogeny of our *B. cereus* strain set.

Seven phylogenetic clusters of *B. cereus* sensu lato have been reported previously [20, 40, 41] and these appear also in our data set. The whole genome analysis reveals that these seven phylogenetic groups are separated by at least 94 % ANI. It has been proposed to use average nucleotide identity between genomes for bacterial species delineation [42]. The current species distinction states that a group of strains belonging to one species must have > 70 % DDH similarity, < 5 °C ΔTm, < 5 % mol G + C difference of total genomic DNA and > 98 % 16S rRNA identity [18]. A boundary of 94–96 % ANI corresponding to ~ 70 % DDH similarity was proposed [42, 43]. Phylogenetic groups within *B. cereus* sensu lato can be distinguished with a species boundary of 94 % identity (Fig. 3). When comparing strains that do not belong to the same phylogenetic group with each other, ANI values are in the range of 80–94 %. Our data therefore suggest that seven genomospecies exist within *B. cereus* sensu lato.

### *B. cereus* sensu lato species affiliation reviewed

The distribution of the strains investigated in this study confirms the observation [12, 17, 20, 21] that many existing species affiliations do not match the genomic relationships (Fig. 1). Cluster IV contains mesophilic pathogenic *B. cereus* and *B. thuringiensis* and one *B. weihenstephanensis* strain. The presence of a unique *cspA* signature is described as being specific for psychrotolerant *B. cereus* sensu lato (*B. mycoides, B. pseudomycoides* and *B. weihenstephanensis*) [20, 44], but the psychrotolerant *cspA-*signature could not be detected in *B. weihenstephanensis* FSL R5-860 (#280, IV). Thus, this strain is likely incorrectly classified as *B. weihenstephanensis*. Cluster III comprises *B. anthracis,* emetic and non-emetic *B. cereus* and *B. thuringiensis*. While it is almost impossible to distinguish between cluster III *B. cereus* and *B. thuringiensis* isolates (Fig. 1), *B. anthracis* is an easily discernable monophyletic group in the species tree. In cluster II potentially pathogenic psychrotolerant strains like *B. cereus* RIVM BC 126 [20] are found that are closely related to cluster III.

However, three phylogenetic groups can be matched easily to three species. Cluster V is clearly separated from all other phylogenetic groups and includes the type strain of the recently described species *B. toyonensis* BCT-7112 which differs from other *B. cereus* sensu lato type strains (ANI < 92 % and a distinct peptidoglycan diamino acid pattern [17]). All isolates belonging to the phylogenetic group V should be renamed as members of *B. toyonensis* [17]. Both *B. toyonensis* BCT-7112 and the newly sequenced *B. cereus* IP5832 are commercially available probiotics, Toyocerin® and Bactisubtil® [36], respectively. Thus, the almost clonally related *Bacillus* strains of cluster V might also be feasible as probiotics for animal feeding but the distribution of enterotoxin genes (see below) probably renders this species also a human pathogen. Cluster I is constituted by *B. pseudomycoides* [20]. Phylogenetic analysis of housekeeping genes clustered *B. mycoides* Rock3-17 (#152, I) together with *B. pseudomycoides* DSM 12442 (type strain, #282, I) in cluster I. Based on these data we suggest that this strain should be renamed as *B. pseudomycoides* after confirmation by fatty acid profiling. Strains of clusters I and VII (*B. cytotoxicus*) show the greatest distance to all other members of *B. cereus* sensu lato.

### Occurrence of virulence genes

To gain an overview over the distribution of virulence genes, 218 *B. cereus* sensu lato strains, including our *de novo* sequenced strains, were analyzed (Additional file 1: Table S2). All *B. cereus* strains possess the *nhe* genes. We analyzed the presence of the toxin genes *hbl, cytK* and *ces* and found that 64 % of the 218 strains contained *hbl* and 34 % of these possess a second *hbl* operon. *CytK-2* appears in 40 %, *cytK-1* in 1 % and the emetic gene cluster *ces* in 5 % of all strains. *CytK-2* is far more frequent in *B. thuringiensis* strains (75 %) than in the rest of *B. cereus* sensu lato (35 %). These results match a study from 2006 that investigated 74 uncharacterized *B. thuringiensis* strains. All of them harbored the *nhe* genes, 74 % *hbl* and 73 % *cytK-2*, displaying about the same potential to cause diarrhea as *B. cereus* [45]. The combined presence of *nhe, hbl* and *cytK* occurs more often among diarrheal (63 %) than among food-borne *B. cereus* sensu stricto strains (33 %) [46]. We find that 30 % of the 218 *B. cereus* sensu lato strains contain all three enterotoxins.

### Evolution of *hbl*

#### Massive horizontal transfer of *hbl*

*Hbl* is not an essential operon, since only 64 % of 218 strains contain *hbl*, which appear rarely in cluster III and are absent from *B. cytotoxicus* (cluster VII) (Fig. 1). A comparison of the concatenated *hblCDAB* gene tree with a species tree consisting of all 101 *hbl*-containing strains shows vastly different topologies (Additional file 2: Figure S2). All phylogenetic clusters except cluster V are mixed which is evidence for massive horizontal transfer of the enterotoxin operon *hblCDAB*, both between and within phylogenetic groups.

It has been speculated that *hblCDAB* is part of a large 18 kb transposon [29, 47, 48]. A detailed comparison of putative transposon regions including *hbl* or *hbl*_*a*_ of the 16 *hbl*-containing strains sequenced *de novo* in this work is shown in Additional file 2: Figure S3. However, while half of the *hbl* operons are inserted within the *uvrC* gene as described earlier [29], in the other strains neither insertion sites nor length of the inserted region or adjacent genes are conserved.

#### Ancient origin of *hblCDA*

**Fig. 4:**
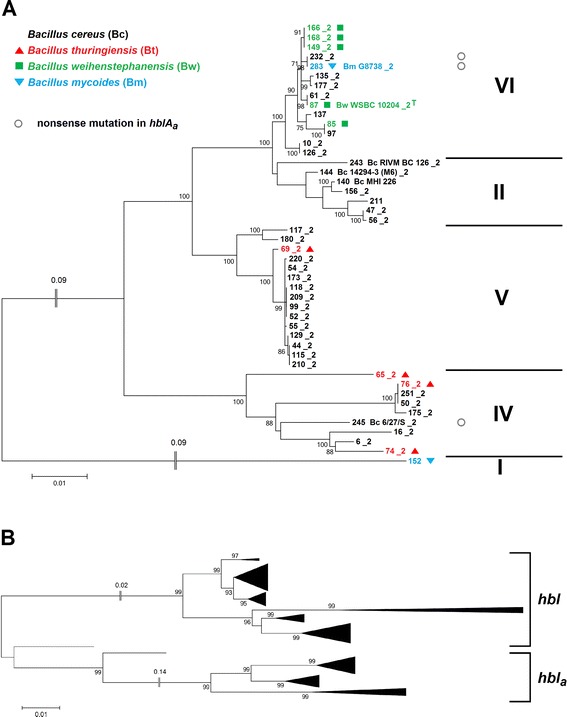
*HblCDA*_*a*_ in *B. cereus* sensu lato. **a**: Phylogenetic *hblCDA*_*a*_ tree (Maximum Likelihood Method) based on the concatenated sequence from 46 *B. cereus* sensu lato strains. **b**: Evidence for an ancient origin of *hbl*_*a*_ based on the concatenated sequence of *hblCDA* and *hblCDA*_*a*_ from 101 *B. cereus* sensu lato strains

Phylogenetic trees represent the species evolution only if orthologous genes are compared which have not been transferred laterally. In contrast, paralogs result from gene duplications [49]. After several generations it becomes increasingly difficult to recognize paralogs, especially when gene loss is involved. It has been known that two distinct homologs of *hbl* exist in *B. cereus* [50]. The more abundant version of *hblCDAB* occurs in 64 % while the second, truncated *hbl*_*a*_ operon (*hblCDA*) is present in 22 % of the investigated strains. Six of the *de novo* sequenced and assembled strains contain two versions of *hbl* and/or *nhe*. We confirmed the existence of duplicated enterotoxin operons by comparison of coverage depths over operons and their respective background contigs as described in (Additional file 1: Tables S5 and S6). The duplication of *hblCDAB* as well as the subsequent loss of *hblB* must be an ancient and unique event which occurred early in the evolution of *B. cereus* sensu lato since all *hblCDA*_*a*_ genes cluster together and are clearly separated from *hblCDA* (Fig. 4b) despite the fact that they are scattered over five phylogenetic clusters. Subsequently, *hblCDA*_*a*_ appears to have been deleted in various lines of the species group (Fig. 1 and Additional file 2: Figure S2). While *hblCDAB* was subject to extensive horizontal gene transfer (see Fig. 1 and Additional file 2: Figure S2), *hblCDA*_*a*_ seems to have been transferred vertically due to a much more conserved tree topology (Fig. 4a and Additional file 2: Figure S2). It has been shown that *hblB* is not essential for enterotoxic activity, because *hblCDAB* mRNA synthesis appears to terminate within the *hblB* gene [51], which might be a pseudogene [52]. *HblCDA*_*a*_ is located on plasmid in the four strains *B. thuringiensis* serovar kurstaki YBT-1520 (pBMB293), *B. thuringiensis* serovar kurstaki HD-1 (pBMB299), *B. thuringiensis* serovar chinensis CT-43 (pCT281) and *B. thuringiensis* serovar thuringiensis IS5056 (pIS56-285), but the detection of plasmid-location may increase with an ongoing completion of genomes.

#### Functional constraints in the evolution of *hbl*

While horizontal transfer of the *hblCDAB* operon is frequent, intra-operon recombinations are extremely rare. Only five statistically significant recombination events within the set of 142 strains could be detected (Additional file 1: Table S4) and all of these occurred exclusively within their respective phylogenetic group. Intra-operon recombinations between different phylogenetic groups have probably been removed by negative selection. DNA sequence identities of *hbl* between *B. cereus* sensu lato strains are quite high (93–100 %). Thus, *hbl* is more conserved than *nhe* or even housekeeping genes in *B. cereus* sensu lato (Figs. 1, 6 and Additional file 2: Figure S2). Both, high sequence conservation and rare intra-operon recombination, suggest that the interaction between Hbl components might be quite specific, thus constraining sequence variation. This assumption is supported by experimental studies of the interaction of Hbl and Hbl_a_ proteins. Both operons encode a functional toxin. However, despite their high similarity, not all Hbl/Hbl_a_ components are exchangeable to form functional toxins [50].

### Horizontal transfer of *cytK*

The third diarrhea causing agent is the single-component chromosomally encoded toxin CytK, which is a hemolytic, dermonecrotic and β-barrel pore-forming enterotoxin [53]. Two variants of CytK are known. 89 % of the amino acid sequence of CytK-2 is identical to CytK-1 and CytK-2 is also able to form pores in planar lipid bilayers, but it shows only 20 % of CytK-1 toxicity [54]. The gene *cytK-2* occurs in 47 % of the strainset and only the two *B. cytotoxicus* strains (#57, #249, VII) possess *cytK-1*. We detected *cytK-2* in strains of clusters II – V, which might have acquired this toxin gene via lateral transfer prior to splitting into clusters II, III and IV (Fig. 1, marked by an arrow) from the *B. cytotoxicus* phylogenetic line where the CytK ancestor may have originated. It may have been lost subsequently in some strains of clusters II and III. In contrast, a recent horizontal transfer of *cytK-2* to a few cluster V strains seems to have occurred (Fig. 1). Lateral transfer of *cytK* can also be inferred from a direct comparison of the *cytK* tree with the species tree of all 68 *cytK*-containing strains (Additional file 2: Figure S4).

### Massive horizontal transfer and sequence variation of the virulence regulator *plcR*

**Fig. 5:**
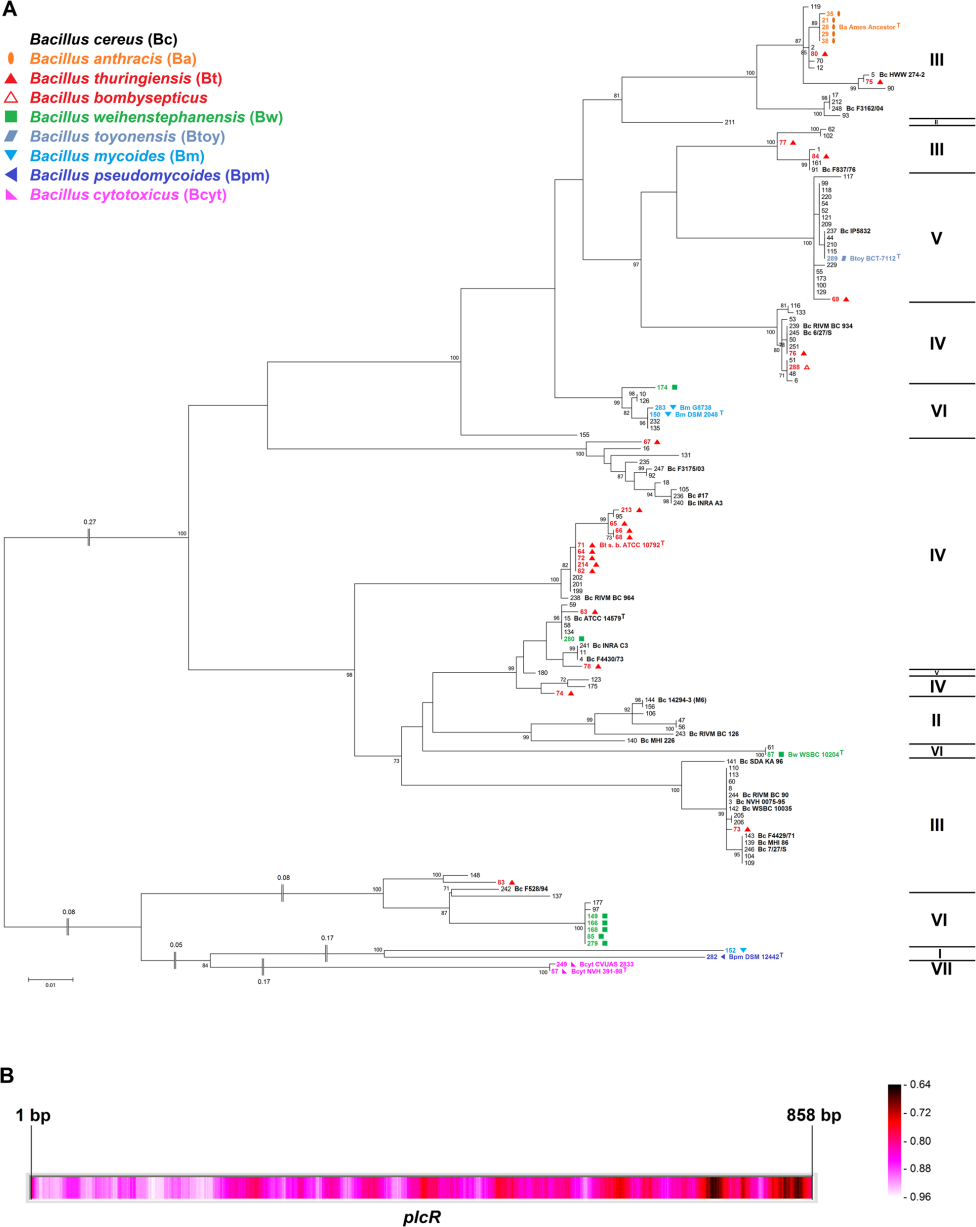
*PlcR* in *B. cereus* sensu lato. **a**: Phylogenetic *plcR* tree (Maximum Likelihood Method) based on the sequence from 142 *B. cereus* sensu lato strains. **b**: Visualization of the sequence homology derived from a multiple *plcR* sequence alignment calculated with RDP3. Color ranges (identity score: ≤ 1, 1 = identical in all sequences) from identical (white) to highly dissimilar (black)

The expression of many virulence factors in *B. cereus* is regulated by the pleiotropic transcriptional activator PlcR (Phospholipase C Regulator) [52]. The promoters of genes regulated by PlcR show a highly conserved palindromic recognition site (TATGNAN_4_TNCATA), which occurs in the promoter regions of *nhe, hbl* [52] and *cytK* [53]. Several other proteins were discovered that are under control of PlcR, such as two-component sensors, chemotaxis proteins, transporters, cytoplasmic regulators and cell wall biogenesis proteins. This indicates that PlcR is a global regulator and a key component in adaptation to (host) environment [55]. The gene phylogeny of *plcR* hints to lateral transfer within 52 *B. cereus, B. anthracis* and *B. thuringiensis* strains [27]. Our comparison of the *plcR* gene phylogeny of 142 strains of all species of the *B. cereus* group (Fig. 5) with the *B. cereus* sensu lato species tree (Fig. 1) confirms a low degree of conservation of *plcR* (DNA sequence identities range from 70 % up to 100 %) and a surprisingly low similarity of both trees. These grossly conflicting topologies may be a result of both frequent horizontal transfer and rapid divergent evolution of *plcR* driven by a variety of environmental selection pressures. The *papR* gene is encoded less than 100 bp downstream of *plcR* and shows a phylogeny highly similar to *plcR* (data not shown). PapR is the quorum sensing peptide necessary for activation PlcR and part of the PlcR regulon [56]. The genomes of *B. pseudomycoides* DSM 12442 (#282, I), *B. mycoides* Rock3-17 (#152, I) and *B. mycoides* Rock1-4 (#151) do not contain *papR*, which might cause reduced pathogenicity of Cluster I strains.

PlcR was experimentally inactivated in *B. cereus* and *B. thuringiensis* by a number of mutations [57] including deletions, additions, nonsense mutations, high diversity, and mutations in *papR* or *opp*, genes necessary for PlcR activation. Inactivation of the PlcR regulon and a subsequent non-hemolytic phenotype also occur naturally. In *B. anthracis* a nonsense mutation after 642 bp leads to a truncated protein, while in *B. cereus* biovar *anthracis* a C-terminal frameshift results in a four amino acids longer protein, both completely inactivate the *plcR* regulon [52, 58]. The *B. cereus* biovar *anthracis plcR* mutation also occurs in *B. cereus* ISP3191 (#102, III). C-terminal *plcR* gene extensions appear in *B. cytotoxicus* (+9 bp), *B. pseudomycoides* (+33 bp) and *B. weihenstephanensis* (+6 bp), but their function is yet unknown. The DNA binding domain helix-turn-helix motif is located in the N-terminal part of the active protein, the regulatory domain in the C-terminal region [59]. The greatest sequence variation is found in the 3′ end of *plcR* (Fig. 5b). While binding to the recognition site requires a highly conserved protein structure, the regulatory function obviously tolerates a higher sequence variation. Hence, this might be a way of allowing fast adaptation to changing environmental or host conditions by modulating transcriptional activation of specific PlcR-controlled genes without unnecessary inactivation of the entire regulon.

### Evolution of *nheABC* enterotoxin genes

#### Ancient origin of *nhe* and recent horizontal gene transfer of the *nhe* variant *nhe*_*a*_

**Fig. 6:**
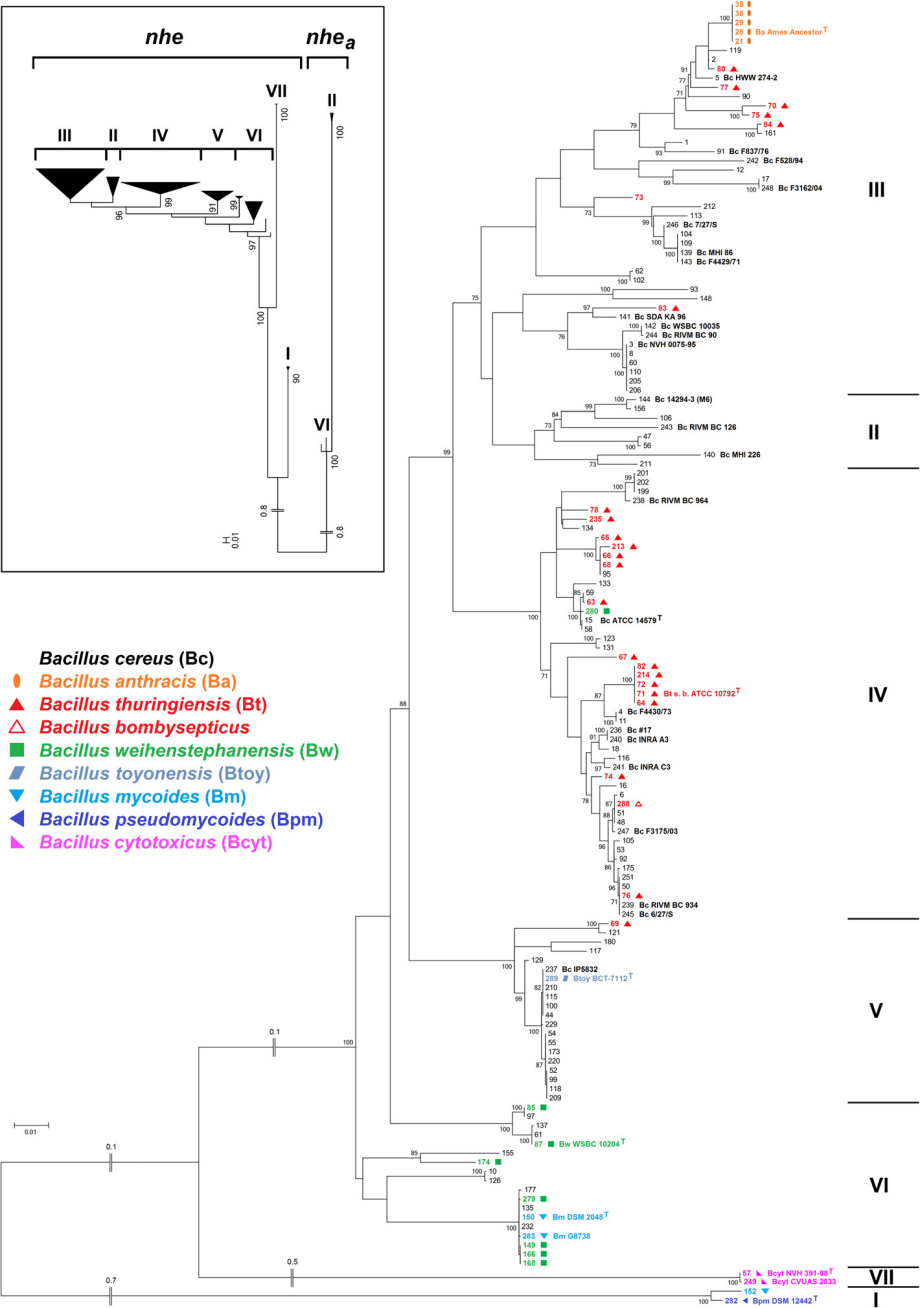
*Nhe* in *B. cereus* sensu lato. Phylogenetic *nheABC* tree (Maximum Likelihood Method) based on the concatenated sequences from 142 *B. cereus* sensu lato strains. Inset: Phylogenetic tree based on the concatenated sequences of *nheABC* and *nheABC*_*a*_ from 142 *B. cereus* sensu lato strains, indicating an ancient origin of *nheABC*_*a*_

**Fig. 7:**
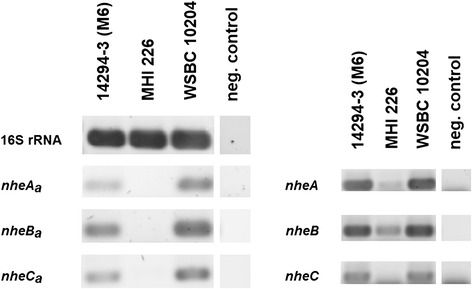
Transcriptional analysis of *nhe* and *nhe*_*a*_ operons. Transcription of *nhe* and *nhe*_*a*_ in *B. cereus* 14294–3 (M6), MHI 226 and *B. weihenstephanensis* WSBC 10204. *Nhe*_*a*_ is transcribed in *B. cereus* 14294–3 (M6) and *B. weihenstephanensis* WSBC 10204, but not in *B. cereus* MHI 226. The latter showed also a weak transcription of *nhe*

While no duplications of *cytK* or *plcR* could be found, a very rare second *nheABC* operon was noticed in four of the 142 *B. cereus* sensu lato strains investigated (Fig. 6). Two *B. cereus* strains (#140, #144, II) and two *B. weihenstephanensis* strains (#85, #87, VI) possess a second *nheABC* variant which we term *nhe*_*a*_. In *B. weihenstephanensis* KBAB4 (#85, VI) the *nhe*_*a*_ operon is part of the 417 kb plasmid pBWB401. The other three *nhe*_*a*_ copies could not be located on a similar plasmid, but were shown to be genuine duplications (Additional file 1: Table S5). The *nhe*_*a*_ operon contains all three *nhe* genes, is actively transcribed, albeit not in all strains (Fig. 7), and we found a putative PlcR recognition site upstream of *nheA*_*a*_ (data not shown). *Nhe*_*a*_ operons differ greatly from all known *nhe* (76–88 % sequence identity), including *B. cytotoxicus’ nheABC* that was until now considered the only major and most distantly related variant. *Nhe*_*a*_ might have resulted from two relatively recent, but separate HGT events into two strains of clusters II and VI, since cluster II and cluster VI *nhe*_*a*_ are two clearly distinct variants. The donor strains harboring the two *nhe*_*a*_ versions have not yet been identified, but sequence comparison shows that their *nhe*_*a*_ must have separated very early in the evolution of the *B. cereus* group (Fig. 6, inset). Apparently, the *nhe*_*a*_ operon is not stably integrated in the genome since several strains in both phylogenetic groups II and VI seem to have lost it shortly after acquisition (Fig. 1). *Nhe*_*a*_ and *hbl*_*a*_ might be starting points for the evolution of new pore-forming enterotoxins in *B. cereus* sensu lato, analogous to the suspected development of *hbl* and *nhe* themselves from an ancient ancestor by gene duplication [48].

### Strictly vertical transmission of *nheABC*

A phylogenetic tree of the concatenated *nheABC* genes was calculated (Fig. 6) and compared with the MLSA species tree (Fig. 1). Surprisingly, their topology is almost identical. Some strains contain more distantly related *nhe* sequences, such as *B. mycoides* and *B. pseudomycoides* (74–77 % sequence identity) or *B. cytotoxicus* NVH 391–98 with only 72–88 % DNA sequence identity to other *nhe* operons [60] (Fig. 6). We suggest that no significant recombination of distantly related *nhe* operons has occurred. After discovering the strictly vertical transmission pattern of *nheABC*, recombination within the *nhe* operon was investigated in more detail. In the set of 142 concatenated *nheABC* genes 21 statistically proven intra-operon recombination events could be detected, but these occurred only between closely related *nhe* regions, which keeps recombination derived variation small (Additional file 1: Table S4). Furthermore, and most significantly, *nhe* occurs in all known *B. cereus* strains without exception, which may be evidence for an important function of the operon. It is noticable that *nheA* is the most highly conserved gene of the *nhe* operon (data not shown).

While Nhe components and their interactions are currently analyzed extensively [61–64] all studies have been focused on the pore-forming activity. However, in NheA an enlarged β-tongue structure was found [64] which, despite its similarity to ClyA and HblB, might be involved in a protein-protein interaction associated with an unknown function. If an unknown function exists, balancing selection may have resulted in a co-evolution of *nhe* with a (yet unknown) interacting factor. Such an interaction would impose certain constraints on the NheA structure, allowing recombination between closely related *nhe* copies but leading to the observed absence of horizontal gene transfer between distantly related strains in order to prevent a fitness loss. We speculate that the strictly vertical evolution, the rare and unstable occurrence of *nhe* copies and high sequence conservation may be evidence for an additional function of Nhe. While the existence of such a hypothetical function can be predicted based on our phylogenetic analysis, further experimental investigation certainly is necessary.

### Evolution of enterotoxin genes and risk assessment

While *B. anthracis* is considered non-enteropathogenic due to the deactivated PlcR-regulon [57], *B. cereus* and *B. thuringiensis* are known to be enterotoxic [48, 65]. *B. weihenstephanensis* strains also carry enterotoxin genes. While this psychrotolerant species is not known to be involved in outbreaks – probably due to reduced toxicity at elevated temperatures [66–68] – it is listed as a risk level 2 organism in Germany. The enterotoxicity of *B. mycoides* and *B. pseudomycoides* is unknown, but our results and other studies confirm that *nhe, hbl* and *cytK* can be found in strains of both species [39, 69, 70]. *B. toyonensis* and other ‘probiotic’ *B. cereus* strains have been used as feed additive. However, their toxic potential is controversially discussed and some are not licensed in the EU [71–73]. Our results confirm the presence of *nhe* and *hbl* in probiotic strains *B. toyonensis* BCT-7112 (#289, V) and *B. cereus* IP5832 (#237, V), as well as *cytK* in two other strains of the species. The pathogenicity of a *B. cereus* sensu lato strain is, most probably, influenced by additional virulence factors such as sphingomyelinase, hemolysin II and metalloproteases [35, 74–76]. Some *B. toyonensis* strains contain *nprA* (neutral protease A), *sph* (sphingomyelinase) and *inhA* (immune inhibitor A) virulence genes. Additional virulence factors can also be found in *B. mycoides* and *B. pseudomycoides* strains. Since HGT is wide-spread among *B. cereus* sensu lato, we consider it difficult to categorize a species of this group as non-pathogenic *per se*. The evolutionary analysis of pathogenicity factors may give reason to initiate a reconsideration of the current enteropathogenicity risk assessment strategies of the whole *B. cereus* group.

## Conclusions

Our results show that seven concatenated housekeeping gene sequences depict species relationships in *B. cereus* sensu lato as accurately as whole-genome comparisons. Many potentially probiotic *B. cereus* strains form a clearly distinguishable phylogenetic line within *B. cereus* sensu lato (Cluster V, ANI boundary > 96 %), which we propose to designate as *B. toyonensis* named according to the type strain. However, species affiliation of strains is contradictory in many cases. The phylogenetic analysis of this study, therefore, calls for a reassessment of this group’s taxonomy. Lateral transfer of virulence genes *hbl, cytK, nhe* and *plcR* within *B. cereus* sensu lato appears to be constrained only by preservation of gene function, which leads us to hypothesize that the strictly vertical transmission of *nhe* operons is caused by a second, unknown but fitness relevant function of *nhe*. We note that evolution of the three *B. cereus* enterotoxin operons is shaped unexpectedly different. Furthermore, ancient diversification of *nhe* and *hbl* operons and propagation of *hbl*_*a*_ suggest a potential to develop new enterotoxin variants. The distribution of pathogenicity factors and frequent recombination among *B. cereus* sensu lato phylogenetic groups should be taken into account during risk assessment of the currently valid species of this group, especially concerning probiotic *B. toyonensis* and *B. mycoides* strains.

## Methods

### *B. cereus* strains and growth conditions

The origin of *Bacillus cereus* sensu lato strains is given in the Additional file 1: Table S2*. B. cereus* strains were grown in either plate count (PC) liquid medium (2.5 g/l yeast extract, 5 g/l peptone from casein and 1 g/l glucose) at 150 rpm or on PC plates at 30 °C. Overnight cultures were inoculated from agar plates or angular agar and grown under shaking (150 rpm) in 3 ml PC medium for 15 h.

### Isolation of genomic DNA

Genomic DNA from *B. cereus* was isolated using a modified CTAB (cetyl trimethylammonium bromide) DNA preparation method [77]. *B. cereus* overnight cultures were pelleted and solved in 567 μl TE buffer (pH 8.0, sterile filtrated). Cells were then lysed using a Fastprep 24 instrument (M. P. Biomedicals, 0.1 mm zirconia beads). Proteins in the supernatant were denatured by adding 30 μl 10 % (w/v) SDS and 3 μl proteinase K (20 mg/ml) and incubated at 37 °C for 3 h. Then, 100 μl 5 M NaCl were added and carefully mixed. 80 μl CTAB/NaCl (10 % CTAB in 0.7 M NaCl) were added, mixed carefully and incubated at 65 °C for 30 min to complex nucleic acids. To separate proteins from nucleic acids phenol chloroform extraction was performed. Nucleic acids were precipitated by adding 0.6 volumes of ice-cold isopropyl alcohol at 4 °C for at least 15 min. Samples were washed twice in ice-cold 70 % (v/v) ethanol, air-dried and dissolved in sterile ddH_2_O at 4 °C overnight. RNA digestion with 20 μg RNAse for 30 min at 37 °C followed by a second phenol chloroform extraction ensured that only genomic DNA remained. Samples were washed twice in ice-cold 70 % (v/v) ethanol, air-dried and dissolved in sterile ddH_2_O at 4 °C overnight. Quality of DNA isolation was tested by agarose gel electrophoresis and spectroscopy (Nanodrop photometer). DNA concentration was determined using the Qubit dsDNA High Sensitivity Assay (Qubit® 2.0 fluorometer).

### NGS sample preparation and sequencing

DNA libraries were prepared using the TruSeq® DNA PCR-Free Sample Preparation Kit. The TruSeq® protocol was optimized with respect to DNA shearing and fragment size selection to improve assembly quality (Huptas *et al.,* in preparation). DNA libraries were sequenced on the Illumina MiSeq® system according to manufacturer’s instructions. The reagent kits used for sequencing of respective strains are indicated in Additional file 1: Table S1.

### Quality control of read data and assembly

Quality of raw sequencing data was assessed by FastQC (www.bioinformatics.bbsrc.ac.uk/projects/fastqc/), followed by quality filtering and trimming of reads with the NGS QC Toolkit v.2.3.2 [78]. Individual settings for each sequenced genome are summarized in Additional file 1: Table S1. The program KmerGenie v.1.5924 [79] was used to calculate the best k-mer value for each assembly from k = 23 to the maximal possible k = 223 in the default two passes. Contigs produced by the short read sequence assembler ABySS v.1.3.7 [80] (minimum scaffold and contig length 500 bp) were further quality controlled by QUAST v.2.2 [81] (using only contigs > 500 bp). Results of data processing, coverage, k-mer values and contig assembly are shown in Additional file 1: Table S1.

### Identification of virulence genes in *de novo* sequenced genomes

The newly generated contigs were aligned against reference genomes (*B. cereus* ATCC 14579, *B. cereus* F837/76 and *B. cytotoxicus* NVH 391/98) with progressiveMauve v.2.3.1 [82] and *nheABC, hblCDAB, cytK, plcR, adk, ccpA, glpF, glpT, panC, pta* and *pycA* were identified according to already annotated features. A second comparison of single contigs with CloneManager Suite 7 (Sci-Ed Software) was used to confirm gene locations and to control start, end and length of genes of interest.

### Collection of data from databases

Further gene sequences and genomes were downloaded from NCBI and Patric databases All available *B. cereus* sensu lato genomic sequences were scanned for the *nhe* operon. Since *B. anthracis* is known to be highly clonal we decided to include only five representative *B. anthracis* strains. A total of 218 strains containing *nheABC* were found and further analyzed for the presence of seven housekeeping genes. The genes *adk* (adenylate kinase), *ccpA* (catabolite control protein A), *glpF* (glycerol uptake facilitator), *glpT* (glycerol-3-phosphate transporter), *panC* (pantoate-β-alanine ligase), *pta* (phosphate acetyltransferase) and *pycA* (pyruvate carboxylase) were chosen to calculate the species tree. These housekeeping genes have already been selected for MLST [39], because they are scattered over the entire chromosome (http://mlstoslo.uio.no/cgi-bin/mlstdb/mlstdbnet4.pl?dbase=optimized&page=scheme-optimized&file=bcereusgrp_isolates.xml). Due to draft status and partially insufficient sequence quality of genome sequences from the databases, selected housekeeping gene sequences could not be identified for all 218 *B. cereus* sensu lato strains. Hence, the final set of strains was reduced to 142 *B. cereus* sensu lato strains (Additional file 1: Table S2). Sequences of the final set are available in the NCBI database either completed or as draft genome. Accession numbers for the 25 genomes reported in this work are given in Additional file 1: Table S1.

### Phylogenetic relationships based on single gene and whole genome sequences

Pairwise average nucleotide identity (ANIb) of 142 *B. cereus* sensu lato genomes was calculated for all possible distinct pairs according to the algorithm described before [43]. For calculation the script ANI.pl (by Jiapeng Chen) available at https://github.com/chjp/ANI was used taking one strain of a pair as query and the other one as reference and vice versa. The resulting two ANIb values for each pair were averaged, which yielded 10,011 similarity values. Similarities were converted into distances by applying the formula 1 – (pairwise avg. ANIb/100). The resulting distance matrix in nexus file format served as input for the neighbor-network computed by SplitsTree4 (version 4.13.1) [83]. Single nucleotide polymorphisms (SNPs) were detected in the entire genomes of the *B. cereus* sensu lato strain set using the program kSNP3 v.3.0 [84] at k-mer size 21 (determined by Kchooser, implemented in kSNP3). The most distant cluster, the two *B. cytotoxicus* strains (phylogenetic cluster VII) had to be excluded to obtain an FCK (fraction of k-mers present in all genomes) value > 0.1, which is necessary for adequate SNP detection efficiency. The resulting core SNP matrix was basis for the calculation of a phylogenetic tree using MEGA6 [85] (Maximum likelihood method, Tamura-Nei model [86], uniform rates, using all sites, bootstrap 1000).

Multiple DNA sequence alignments were generated online with ClustalΩ [87] and used to compare genes. Alignments of (concatenated) genes served as input for MEGA6. The species tree of *B. cereus* sensu lato strains was built from concatenated DNA sequences of seven housekeeping genes. The genes *adk, ccpA, glpF, glpT, panC, pta,* and *pycA* were taken from the optimized MLST scheme developed by Tourasse *et al.* [39], because they are evenly distributed over the entire *B. cereus* chromosome. In contrast to the MLST-approach, we decided to use the entire genes in our comparison to display relationships as accurately as possible. To compare the operons *nheABC* and *hblCDAB* concatenated sequences without intergenic regions were used. The order of the genes within the operons was conserved in all strains, thus a rearrangement of genes was not necessary. Phylogenetic trees of genes or concatenated genes were calculated in MEGA6 using the maximum likelihood method based on the Tamura-Nei model with a discrete Gamma distribution, permitting some invariant sites (TN93 + G + I). This was the ideal substitution model determined by the ‘Find Best DNA/Protein Models (ML)’ function of MEGA6 [85] for the species tree dataset containing seven concatenated housekeeping genes of 142 strains and was applied for the calculation of all phylogenetic trees. Sequences were first aligned and then shortened to the ‘lowest common denominator’ to ensure that all sequences of an alignment start and end together, because protruding ends within an alignment are automatically cut during calculation of the phylogenetic tree (gaps within sequences remained intact). While the housekeeping genes were of the same length in all strains, the toxin genes did not show uniform lengths in alignments and required cutting of sequence ends. All sites (including gaps) were used to calculate the phylogenetic trees and reliability was tested by 1000 bootstrap repeats. Branching points with bootstrap values ≥ 70 are considered reliable. The phylogenetic trees are not rooted to a certain outgroup to allow comparison of subsets and toxin genes that only occur in some strains. To ensure comparability, we used the same settings for the calculation of all phylogenetic trees. To further control the reliability of the maximum likelihood approach, the phylogenetic tree calculations were repeated using the neighbor joining and minimum evolution methods implemented in MEGA6. Results were almost identical albeit with lower resolutions (data not shown).

### Detection of potential recombination events

Putative recombination events within enterotoxin operons and a graphical representation of differences within multiple alignments were calculated by RDP3 [88]. In a very conservative approach only statistically proven recombination events according to the following criteria were considered: a recombination event i) was detected by at least three of the programs implemented in RDP3, ii) showed a maximum average p-value of < 0.05 and iii) both parental sequences are part of the investigated strainset.

### Analysis of *nhe* and *nhe*_*a*_ transcription

Bacteria were cultured in CGY medium [89] supplemented with 1 % (w/v) glucose as described previously [35] to an OD_600_ of 4. Six ml of the culture were harvested, the cell pellets were snap-frosted in liquid nitrogen and stored at −80 °C. Total RNA was extracted by resuspending the pellet in 1 ml TRIreagent and cells were disrupted as described above. DNase I digestion and RNA isolation were performed as previously described [90]. First strand synthesis was performed using the qScript™ cDNA Supermix (Quanta Biosciences). Subsequent PCR (annealing temperature 56 °C) contained 50 pmol of each primer (Additional file 1: Table S3), 5 μl green GoTaq® G2 reaction buffer, 0.05 mM of each dNTP, 62.5 mM MgCl_2_ and 0.5 U GoTaq® G2 DNA Polymerase (Promega) in a volume of 25 μl. Amplification of the 16S rRNA gene *rrn* transcript served as a positive control, nuclease-free H_2_O as a negative control. RT-PCR results were visualized on 2 % (w/v) agarose gels.

## Availability of supporting data

Draft genome assemblies are deposited in NCBI’s GenBank, accession numbers are listed in Additional file 1: Table S1. All data supporting the results of this article are included within the article and its Additional files 1 and 2.

## Additional files

Additional file 1:
**Contains Tables S1-S6.** (PDF 561 kb) Additional file 2:
**Contains Figures S1-S4.** (PDF 1084 kb)

## Abbreviations

*Adk*: Gene encoding adenylate kinase
ANI: Average nucleotide identity
ANIb: Calculation of ANI based on the BLAST algorithm
AFLP: Amplified fragment length polymorphism
*Cap*: Operon encoding *B. anthracis* poly-γ-D-glutamic acid capsule
*CcpA*: Gene encoding catabolite control protein A
*Ces*: Seven gene operon encoding among others both cereulide synthetase subunits A and B
*ClyA*: Gene encoding the pore-forming hemolysin cytolysin A
*Cry*: Genes encoding *B. thuringiensis* insecticidal crystal endotoxins
*CspA*: Gene encoding cold shock protein A
*Cya*: Gene encoding *B. anthracis* edema factor
*Cyt*: Genes encoding *B. thuringiensis* cytolysins
*CytK*: Gene encoding cytotoxin K, exists in the variants CytK-1 and CytK-2
DDH: DNA-DNA Hybridization
FCK: Fraction of core k-mers
*GlpF*: Gene encoding glycerol uptake facilitator protein
*GlpT*: Gene encoding glycerol-3-phosphate transporter
*Hbl*: Operon encoding the tripartite enterotoxin hemolysin BL, exists in the variants Hbl and Hbl_a_
HGT: Horizontal gene transfer
*Lef*: Gene encoding *B. anthracis* lethal factor
ML: Maximum likelihood
MLEE: Multi locus enzyme electrophoresis
MLSA: Multi locus sequence analysis
MLST: Multi locus sequence typing
*Nhe*: Operon encoding the tripartite non-hemolytic enterotoxin, exists in the variants Nhe and Nhe_a_
*PagA*: Gene encoding *B. anthracis* protective antigen A
*PanC*: Gene encoding pantoate-β-alanine ligase
*PapR*: Gene encoding the quorum sensing peptide PapR necessary for activation of PlcR
*PlcR*: Gene encoding the Phospholipase C virulence regulator
*Pta*: Gene encoding phosphotransacetylase
pXO1: *B. anthracis* virulence mega-plasmid containing the pathogenicity island encoding among others PagA, Lef and Cya
pXO2: *B. anthracis* virulence mega-plasmid containing among others the *cap* operon
*PycA*: Gene encoding pyruvate carboxylase subunit A
SNP: Single nucleotide polymorphism
Tm: Melting temperature
