# Supplementary material for: Massive horizontal gene transfer, strictly vertical inheritance and ancient duplications differentially shape the evolution of Bacillus cereus enterotoxin operons hbl, cytK and nhe
Source: BMC Evol Biol. 2015 Nov 10;15:246. doi: 10.1186/s12862-015-0529-4 (PMC4641410; doi:10.1186/s12862-015-0529-4)
Supplement: Additional file 1: — Contains Tables S1-S6. (PDF 561 kb) [file 12862_2015_529_MOESM1_ESM.pdf]

Table S1: *De novo* whole genome sequencing results of 25 *B. cereus* sensu lato strains.

Sequencing statistics and results of read quality filtering/trimming and contig assembly are reported.

Strain	MiSeq® reagent Kit	Quality filtering	Raw read length [bp]	# Raw reads	# Filtered & trimmed reads	Trimmed read length [bp]	Genome coverage trimmed reads (x-fold)	K-mer size [bp]	N50	# Contigs >500 bp	Assembly size [bp]	NCBI GenBank accession number
B. cereus F4430/73	Version 1, 300 cycles, paired end	Length ≥ 70%, Q ≥ 20	145	2,008,054	1,483,038	113	**32**	47	25,336	**447**	**5,577,793**	JYPK00000000
B. cereus HWW 274-2				2,837,642	1,505,012		**32**	39	32,259	**320**	**5,290,159**	JYPL00000000
B. cereus WSBC 10035	Version 2, 500 cycles, paired end	Length ≥ 70%, Q ≥ 20	180	2,996,320	1,247,284	169	**38**	75	52,377	**184**	**5,619,577**	LABS00000000
B. cereus F4429/71				3,129,982	1,491,644		**45**	67	91,497	**115**	**5,284,967**	JYPJ00000000
B. cereus 14294-3 (M6)	Version 2, 500 cycles, paired end	Length ≥ 80%, Q ≥ 30	200	5,225,132	4,625,090	189	**165**	99	875,707	**25**	**5,523,305**	JYPF00000000
B. cereus INRA A3	Version 3, 600 cycles, paired end	Length ≥ 80%, Q ≥ 30	250	13,534,264	7,187,650	239	**323**	169	189,988	**61**	**6,075,647**	LABH00000000
B. cereus INRA C3				9,841,330	4,702,936		**213**	173	348,619	**38**	**5,596,453**	LABI00000000
B. cereus RIVM BC 934				13,029,356	6,521,168		**295**	203	62,879	**207**	**6,840,916**	LABP00000000
B. cereus RIVM BC 964	Version 3, 600 cycles, paired end	Length ≥ 80%, Q ≥ 30	250	9,914,386	5,338,252	192	**194**	43	136,264	**78**	**5,815,402**	LABQ00000000
B. cereus #17				9,276,110	5,007,666	192	**182**	101	237,244	**45**	**5,852,222**	JYFW00000000
B. cereus IP5832				15,632,892	9,878,046	196	**366**	117	470,309	**28**	**5,592,318**	LABJ00000000
B. weihenstephanensis WSBC 10204				10,434,454	6,063,602	191	**219**	85	296,898	**43**	**5,655,039**	Complete genome available
B. cereus RIVM BC 126	Version 3 600 cycles, paired end	Length ≥ 80%, Q ≥ 30	250	3,742,980	2,960,928	230	**129**	183	340,103	**47**	**5,417,487**	LABO00000000
B. cereus NVH 0075-95				4,453,098	3,407,364	220	**142**	177	172,877	**92**	**6,112,682**	LABM00000000
B. cereus MHI 226				2,800,238	2,248,024	230	**98**	113	180,772	**60**	**6,233,017**	LABL00000000
B. cereus RIVM BC 90				4,690,328	3,816,892	230	**166**	183	166,503	**68**	**5,559,670**	LABN00000000
B. cereus 6/27/S				3,507,602	2,686,982	220	**112**	115	67,058	**176**	**6,771,128**	LABV00000000
B. cereus SDA KA 96				4,266,240	3,498,326	220	**146**	167	402,005	**34**	**5,335,844**	LABR00000000
B. cereus 7/27/S				3,604,194	2,965,452	225	**126**	153	259,768	**48**	**5,479,572**	LABW00000000
B. cereus MHI 86				3,970,862	3,038,394	220	**126**	63	192,413	**54**	**5,551,873**	LABK00000000
B. cereus F3175/03				2,913,134	2,235,522	220	**93**	73	204,296	**64**	**5,733,808**	JYPI00000000
B. cereus F3162/04				3,033,036	2,338,464	220	**97**	43	170,740	**77**	**5,591,156**	JZEV00000000
B. cytotoxicus CVUAS 2833				3,185,212	2,298,738	210	**91**	177	318,402	**36**	**4,127,075**	JYPG00000000
B. cereus F528/94	Version 3, 600 cycles, paired end	Length ≥ 80%, Q ≥ 30	230	7,027,494	5,982,372	219	**248**	167	355,056	**49**	**5,935,300**	JYPH00000000
B. mycoides WSBC 10969				6,040,358	4,819,360	210	**192**	167	270,494	**54**	**6,101,972**	LABT00000000

Reference genome: *B. cereus* F837/76 (5,288,498 bp)

Table S2: List of 218 *B. cereus* sensu lato strains investigated in this study.

142 strains listed in the first part of the table were included in the final set and are listed according to their cluster affiliation (C1 – C7) derived from concatenated housekeeping gene species tree (Fig. 1). Presence (x) or absence (-) of toxin and regulator genes isindicated. For 76 strains listed in the second part of the table, one or more housekeeping gene(s) or *plcR* could not be identified from the genome sequence. These strains were excluded from further analyses. Type strains are highlighted in bold, strains sequenced in this study are marked by an asterisk.

Ba: *Bacillus anthracis*, Bb: *Bacillus bombysepticus*, Bcyt: *Bacillus cytotoxicus*, Bm: *Bacillus mycoides*, Bpm: *Bacillus pseudomycoides*, Bt: *Bacillus thuringiensis*, Btoy: *Bacillus toyonensis*, Bw : *Bacillus weihenstephanensis*, not specified name: *Bacillus cereus*.

Part 1: Final strainset

Cluster	Strain	Original name	*nhe*	*hbl*	*cytK*	*plcR*	2. *hbl*	2. *nhe*	*ces*	Source	Reference
**Cluster 1 (C1)**											
C1_S1	282	**Bpm DSM 12442**	x	-	-	x	-	-	-	Soil	[1]
C1_S2	152	Bm Rock3-17	x	-	-	x	x	-	-	Soil, Maryland	[2]
**Cluster 2 (C2)**											
C2_S1	47	BAG2X1-2	x	x	-	x	x	-	-	Soil, Massachusetts	[3]
C2_S2	56	BAG6X1-1	x	x	-	x	x	-	-	Soil, Massachusetts	[3]
C2_S3	106	MM3	x	x	-	x	-	-	-	Food	[2]
C2_S4	140	MHI 226 *	x	-	-	x	x	x	-	Milk and milk products, Germany	1)
C2_S5	144	14294-3 (M6) *	x	x	x	x	x	x	-	Ice cream, Germany, 2004	3) WSBC 10904
C2_S6	156	BAG5X2-1	x	x	x	x	x	-	-	Soil, Massachusetts	[3]
C2_S7	211	BAG2O-3	x	-	-	x	x	-	-	Soil, Massachusetts	[3]
C2_S8	243	RIVM BC 126 *	x	x	-	x	x	-	-	Patients faeces, Netherlands, 1999	2)
**Cluster 3 (C3)**											
C3_S1	28	**Ba str. Ames Ancestor**	x	-	-	x	-	-	-	Dead heifer, Texas	[4]
C3_S2	29	Ba str. Ames	x	-	-	x	-	-	-	Laboratory strain	[5]
C3_S3	21	Ba str. A0248	x	-	-	x	-	-	-	Human, USAMRIID, Ohio	sequenced by J. Craig Venter Institute
C3_S4	35	Ba str. H9401	x	-	-	x	-	-	-	Clinical, cutaneous anthrax, Korea	[6]
C3_S5	38	Ba str. Sterne	x	-	-	x	-	-	-	Laboratory strain	[4]
C3_S6	1	03BB102	x	-	-	x	-	-	-	Dust, fatal pneumonia, Texas	Sequenced by TIGR
C3_S7	2	95/8201	x	-	x	x	-	-	-	Endocarditis, UK 1995	[2]

C3_S8	3	NVH 0075-95 *	x	-	-	x	-	-	-	Stew with vegetables, food poisoning, Norway, 1995	[7]
C3_S9	5	HWW 274-2 *	x	-	x	x	-	-	-	Milk powder, Germany, 2004	Lang-Halter, unpublished
C3_S10	8	AH187	x	-	-	x	-	-	x	Vomit, cooked rice, London 1972	[8]
C3_S11	12	AH820	x	x	x	x	-	-	-	Periodontosis, Norway 1995	[9]
C3_S12	17	ATCC 10987	x	-	x	x	-	-	-	Cheese spoilage, Canada 1930	[10]
C3_S13	60	BDRD-ST26	x	-	-	x	-	-	-	BDRD stock strain	[2]
C3_S14	62	biovar anthracis str. Cl	x	-	-	x	-	-	-	Chimpanzee, fatal anthrax, Cote d'Ivoire (CI) 2001	[11]
C3_S15	70	Bt serovar andalousiensis BGSC 4AW1	x	-	x	x	-	-	-	Spain	[2]
C3_S16	73	Bt serovar finitimus YBT-020	x	x	-	x	-	-	-	Soil, China (Huazhong Agricultural University)	[12]
C3_S17	75	Bt serovar konkukian str. 97-27	x	x	x	x	-	-	-	Wound infection, french soldier in Yugoslawia	[13]
C3_S18	77	Bt serovar monterrey BGSC 4AJ1	x	x	x	x	-	-	-	Mexico	[14]
C3_S19	80	Bt serovar pulsiensis BGSC 4CC1	x	-	x	x	-	-	-	Grain field, Pakistan	[2]
C3_S20	83	Bt serovar tochigiensis BGSC 4Y1	x	x	x	x	-	-	-	Soil, Japan	[2]
C3_S21	84	Bt str. Al Hakam	x	x	-	x	-	-	-	Suspected bioweapon facility, Iraq	[15]
C3_S22	90	E33L	x	-	x	x	-	-	-	Zebra carcass, Namibia, 1996	[4]
C3_S23	91	F837/76	x	x	-	x	-	-	-	Human, wound, postoperative infection	[16]
C3_S24	93	FRI-35	x	-	x	x	-	-	-	?	Sequenced by Los Alamos National
C3_S25	102	ISP3191	x	-	-	x	-	-	-	Spice, Belgium	[17]
C3_S26	104	m1293	x	-	-	x	-	-	-	Cream cheese	[2]
C3_S27	109	MSX-D12	x	-	x	x	-	-	-	Antarctic concordia station and ISS	[3]
C3_S28	110	NC7401	x	-	-	x	-	-	x	Food poisoning, chow mein, Japan	[18]
C3_S29	113	Q1	x	-	-	x	-	-	-	Deep surface oil reseroir, China	[19]
C3_S30	119	Rock3-42	x	-	x	x	-	-	-	Soil, Rockville in Maryland	[2]
C3_S31	139	MHI 86 *	x	-	x	x	-	-	-	Infant food, Germany	1)
C3_S32	141	SDA KA 96 *	x	x	x	x	-	-	-	Raw milk, Sweden, 1997	[20]
C3_S33	142	WSBC 10035 *	x	-	-	x	-	-	-	Pasteurized milk, Germany, 1993	[20]
C3_S34	143	F4429/71 *	x	-	x	x	-	-	-	Vanilla pudding, Netherlands, 1971	1) MHI 1543
C3_S35	148	ATCC 4342	x	x	-	x	-	-	-	?	[2]
C3_S36	161	BGSC 6E1	x	x	-	x	-	-	-	?	[2]
C3_S37	205	IS195	x	-	-	x	-	-	x	Intestine of bank vole, Poland	[21]
C3_S38	206	IS845/00	x	-	-	x	-	-	-	Intestine of bank vole, Poland	[21]
C3_S39	212	F	x	-	-	x	-	-	-	Permafrost sample, 3 mio. years old	Institute of chemical biology and fundamental
C3_S40	242	F528/94 *	x	x	-	x	-	-	-	Beef chow mein & rice, food poisoning, UK	[22]
C3_S41	244	RIVM BC 90 *	x	-	-	x	-	-	-	Human faeces, Netherlands, 1999	2)
C3_S42	246	7/27/S *	x	-	-	x	-	-	-	Human faeces	1) MHI 3185

C3_S43	248	F3162/04 *	x	-	x	x	-	-	-	Human faeces, 2004	1) MHI 3173
**Cluster 4 (C4)**											
C4_S1	15	**ATCC 14579**	x	x	x	x	-	-	-	Air, cow-shed	[23]
C4_S2	71	**Bt serovar berliner ATCC 10792**	x	x	x	x	-	-	-	Mediterranean flour moth (Ephestia kuehniella)	[2]
C4_S3	4	F4430/73 *	x	x	x	x	-	-	-	Pea soup, Belgium, 1973	[24]
C4_S4	6	172560W	x	x	x	x	x	-	-	Burn wound	[2]
C4_S5	11	AH676	x	x	x	x	-	-	-	Soil, Norway	[2]
C4_S6	16	ATCC 10876	x	x	x	x	x	-	-	?	[2]
C4_S7	18	B4264	x	x	x	x	-	-	-	Fatal pneumonia, blood and pleural fluid, 1969	[2]
C4_S8	48	BAG3O-2	x	x	x	x	-	-	-	Soil, Massachusetts	[3]
C4_S9	50	BAG3X2-2	x	x	x	x	x	-	-	Soil, Massachusetts	[3]
C4_S10	51	BAG4O-1	x	x	x	x	-	-	-	Soil, Massachusetts	[3]
C4_S11	53	BAG4X12-1	x	x	x	x	-	-	-	Soil, Massachusetts	[3]
C4_S12	58	BDRD-Cer4	x	x	x	x	-	-	-	BDRD stock strain	[2]
C4_S13	59	BDRD-ST24	x	x	x	x	-	-	-	BDRD stock strain	[2]
C4_S14	63	Bt BMB171	x	x	x	x	-	-	-	Lab strain, China	[25]
C4_S15	64	Bt Bt407	x	x	x	x	-	-	-	? Strain isolated by O. Arantes	[26]
C4_S16	65	Bt HD-771	x	x	-	x	x	-	-	?	Sequenced by Los Alamos National
C4_S17	66	Bt HD-789	x	x	x	x	-	-	-	?	Sequenced by Los Alamos National
C4_S18	67	Bt IBL 200	x	x	x	x	-	-	-	Human	[2]
C4_S19	68	Bt IBL 4222	x	x	x	x	-	-	-	Cat	[2]
C4_S20	72	Bt serovar chinensis CT-43	x	x	x	x	-	-	-	China	[27]
C4_S21	74	Bt serovar huazhongensis BGSC 4BD1	x	x	-	x	x	-	-	China	[2]
C4_S22	76	Bt serovar kurstaki str. T03a001	x	x	x	x	x	-	-	Mediterranean flour moth (Ephestia kuehniella)	[2]
C4_S23	78	Bt serovar pakistani str. T13001	x	x	x	x	-	-	-	Lepidoptera	[2]
C4_S24	82	Bt serovar thuringiensis str. T01001	x	x	x	x	-	-	-	Mediterranean flour moth (Ephestia kuehniella)	[2]
C4_S25	92	F65185	x	x	x	x	-	-	-	Open fracture, New York	[2]
C4_S26	95	G9842	x	x	-	x	-	-	-	Stool, food poisoning, Nebraska 1996	[28]
C4_S27	105	m1550	x	x	x	x	-	-	-	Uncooked chicken, Brazil	[2]
C4_S28	116	Rock1-15	x	x	x	x	-	-	-	Soil, Rockville in Maryland	[2]
C4_S29	123	VD014	x	x	x	x	-	-	-	Soil, Spain	[17]
C4_S30	131	VD156	x	x	x	x	-	-	-	Soil, Abu Dhabi, UAE	[17]
C4_S31	133	VD169	x	x	x	x	-	-	-	Dubai, UAE	[17]
C4_S32	134	VD200	x	x	x	x	-	-	-	Water, Scotland	[17]
C4_S33	175	VD133	x	x	x	x	x	-	-	Soil, Martinique	[17]

C4_S34	199	BAG2O-1	x	-	x	x	-	-	-	Soil, Massachusetts	[3]
C4_S35	201	BAG1X2-2	x	-	x	x	-	-	-	Soil, Massachusetts	[3]
C4_S36	202	BAG1X2-1	x	-	x	x	-	-	-	Soil, Massachusetts	[3]
C4_S37	213	Bt DAR 81934	x	x	x	x	-	-	-	Australia	[29]
C4_S38	214	Bt serovar thuringiensis str. IS5056	x	x	x	x	-	-	-	Soil, Biebrza Nation Park, Poland	[30]
C4_S39	235	Bt YBT-1518	x	x	x	x	-	-	-	Soil, China	Huazhong Agricultural University
C4_S40	236	#17 *	x	x	x	x	-	-	-	Mouse microbiota	Obtained from Thomas Clavel
C4_S41	238	RIVM BC 964 *	x	-	x	x	-	-	-	Kebab, Netherlands, 2002	2)
C4_S42	239	RIVM BC 934 *	x	x	x	x	-	-	-	Lettuce, Netherlands, 2002	[20]
C4_S43	240	INRA A3 *	x	x	x	x	-	x	-	Starch, Normandie, France, 1998	[20]
C4_S44	241	INRA C3 *	x	x	x	x	-	-	-	Pasteurized carrot, Vaucluse, France, 1996	[20]
C4_S45	245	6/27/S *	x	x	x	x	x	-	-	Human faeces	1) MHI 3172
C4_S46	247	F3175/03 *	x	x	x	x	-	-	-	Human faeces, 2004	1) MHI 3169
C4_S47	251	HD73	x	x	x	x	x	-	-	?	Sequenced by Broad Institute
C4_S48	280	Bw FSL R5-860	x	x	x	x	-	-	-	Pasteurized Milk	Sequenced by Cornell University
C4_S49	288	Bb str. Wang	x	x	x	x	-	-	-	Cadaver of silkworm larvae (Bombyx mori)	[31]
**Cluster 5 (C5)**											
C5_S1	289	**Btoy BCT-7112**	x	x	-	x	-	-	-	Purified for use as probiotic, Japan 1966	[32]
C5_S2	44	BAG1O-2	x	x	-	x	x	-	-	Soil, Massachusetts	[3]
C5_S3	52	BAG4X2-1	x	x	-	x	x	-	-	Soil, Massachusetts	[3]
C5_S4	54	BAG5O-1	x	x	-	x	x	-	-	Soil, Massachusetts	[3]
C5_S5	55	BAG6O-1	x	x	-	x	x	-	-	Soil, Massachusetts	[3]
C5_S6	69	Bt MC28	x	x	-	x	x	-	-	Forest, Sichuan China	[33]
C5_S7	99	HuB2-9	x	x	-	x	x	-	-	Environmental isolate	[17]
C5_S8	100	HuB5-5	x	x	-	x	-	-	-	Environmental isolate	[17]
C5_S9	115	Rock1-3	x	x	-	x	x	-	-	Soil, Rockville in Maryland	[2]
C5_S10	117	Rock3-28	x	x	-	x	x	-	-	Soil, Rockville in Maryland	[2]
C5_S11	118	Rock3-29	x	x	-	x	x	-	-	Soil, Rockville in Maryland	[2]
C5_S12	121	Rock4-18	x	x	x	x	-	-	-	Soil, Rockville in Maryland	[2]
C5_S13	129	VD148	x	x	-	x	x	-	-	Soil, Switzerland	[17]
C5_S14	173	HuB4-10	x	x	-	x	x	-	-	Environmental isolate	[17]
C5_S15	180	VD115	x	x	x	x	x	-	-	Soil, France	[17]
C5_S16	209	VD214	x	x	-	x	x	-	-	Water, Scotland	[17]
C5_S17	210	BAG2O-2	x	x	-	x	x	-	-	Soil, Massachusetts	[3]
C5_S18	220	HuA2-3	x	x	-	x	x	-	-	Environmental isolate	[17]

C5_S19	229	VD131	x	x	-	x	x	-	-	Soil, Martinique	[17]
C5_S20	237	IP5832 *	x	x	-	x	-	-	-	Commercial probiotic	[34]
**Cluster 6 (C6)**											
C6_S1	87	**Bw WSBC 10204 ***	x	x	-	x	x	x	-	Milk	[35]
C6_S2	150	**Bm DSM 2048**	x	x	-	x	-	-	-	Soil	[2]
C6_S3	10	AH621	x	x	-	x	x	-	-	Soil, Norway	[2]
C6_S4	61	BDRD-ST196	x	x	-	x	x	-	-	BDRD stock strain	[2]
C6_S5	85	Bw KBAB4	x	-	-	x	x	x	-	Soil	[4]
C6_S6	97	HuA2-4	x	-	-	x	x	-	-	Environmental isolate	[17]
C6_S7	126	VD048	x	x	-	x	x	-	-	Soil, Denmark	[17]
C6_S8	135	VDM022	x	x	-	x	x	-	-	Soil, Greenland	[17]
C6_S9	137	VDM062	x	-	-	x	x	-	-	Soil, Scotland	[17]
C6_S10	149	Bw BtB2-4	x	x	-	x	x	-	-	Forest soil, Belgium	[36]
C6_S11	155	BAG5X1-1	x	x	-	x	-	-	-	Soil, Massachusetts	[3]
C6_S12	166	Bw CER057	x	x	-	x	x	-	x	Parsley, Belgium	[36]
C6_S13	168	Bw CER074	x	x	-	x	x	-	x	Raw milk, Belgium	[36]
C6_S14	174	Bw MC67	x	x	-	x	-	-	x	Soil, Denmark	[37]
C6_S15	177	VD078	x	x	-	x	x	-	-	Soil, Greenland	[17]
C6_S16	232	VDM019	x	x	-	x	x	-	-	Soil, Greenland	[17]
C6_S17	279	Bw FSL H7-687	x	x	-	x	-	-	-	Pasteurized Milk	Sequenced by Cornell University
C6_S18	283	Bm WSBC 10969 *	x	x	-	x	x	-	-	Raw milk, Germany, 2014	This study
**Cluster 7 (C7)**											
C7_S1	57	**Bcyt NVH 391-98**	x	-	x	x	-	-	-	Vegetable puree, food poisoning, France, 1998	[38]
C7_S2	249	Bcyt CVUAS2833 *	x	-	x	x	-	-	-	Potato puree, food poisoning, Germany, 2007	[39]

1) Strain collection of the Department for Hygiene and Technology of Milk (MHI), Germany
2) Strain collection of the Rijksinstituut voor Volksgezondheid en Milieu (RIVM), Netherlands
3) Weihenstephan *Bacillus cereus* group Strain Collection (WSBC), Germany

Part 2: Additional strains

Cluster	Strain	Original name	*nhe*	*hbl*	*cytK*	*plcR*	2. *hbl*	2. *nhe*	*ces*	Source	Reference
	9	AH603	x	x	-	-	-	-	-	Dairy	[2]
	13	AH1134	x	x	x	-	-	-	-	Pediatric endophtalmitis, Oklahoma City, Dean	Sequenced by TIGR
	14	AND1407	x	-	-	-	-	-	x	Blackcurrant	[36]
	19	Ba str. A0174	x	-	-	-	-	-	-	Canada	Sequenced by Los Alamos National
	20	Ba str. A0193	x	-	-	-	-	-	-	Bovine isolate, South Dakota	Sequenced by Los Alamos National
	22	Ba str. A0389	x	-	-	-	-	-	-	Bekasi, Indonesia	Sequenced by Los Alamos National
	23	Ba str. A0442	x	-	-	-	-	-	-	Kudu (Antelope), Kruger National Park, South	Sequenced by Los Alamos National

24	Ba str. A0465	x	-	-	-	-	-	-	Bovine isolate, France	Sequenced by Los Alamos National
25	Ba str. A0488	x	-	-	-	-	-	-	Infected cattle, UK 1935	Sequenced by Los Alamos National
26	Ba str. A1055	x	-	-	-	-	-	-	From Paul Keim´s laboratory	[4]
27	Ba str. A2012	x	-	-	-	-	-	-	Clinical, inhalational anthrax, West Palm	[5]
30	Ba str. Australia 94	x	-	-	-	-	-	-	Australia	[4]
31	Ba str. BF1	x	-	-	-	-	-	-	Cow carcass, Bavaria, Germany	[40]
32	Ba str. CDC 684	x	-	-	x	-	-	-	?	Sequenced by J. Craig Venter Institute
33	Ba str. CNEVA-9066	x	-	-	-	-	-	-	France	[4]
34	Ba str. Carbosap	x	-	-	-	-	-	-	Italy	[41]
36	Ba str. Heroin Ba4599	x	-	-	-	-	-	-	Clinical, first case of anthrax outbreak,	[42]
37	Ba str. Kruger B	x	-	-	-	-	-	-	Kruger National Park, Sout Africa	[5]
39	Ba str. Tsiankovskii-I	x	-	-	-	-	-	-	Soviet Union	Sequenced by J. Craig Venter Institute
40	Ba str. UR-1	x	-	-	-	-	-	-	Clinical, injectional anthrax in a German	[43]
41	Ba str. Vollum	x	-	-	-	-	-	-	Occurs in the UK, Spain, Zimbabwe	[4]
42	Ba str. Western North America USA6153	x	-	-	-	-	-	-	?	[4]
45	BAG1X1-2	x	x	x	-	-	-	-	Soil, Massachusetts	[3]
46	BAG1X1-3	x	x	-	x	-	-	-	Soil, Massachusetts	[3]
49	BAG3X2-1	x	x	-	x	-	-	-	Soil, Massachusetts	[3]
79	Bt serovar pondicheriensis BGSC	x	x	x	x	-	-	-	Soil, India	[2]
81	Bt serovar sotto str. T04001	x	x	-	x	x	-	-	Canada	[2]
94	G9241	x	x	x	-	-	-	-	Pneumonia, 1987	[44]
96	Bt s. kurstaki str. HD73	x	x	x	x	x	-	-	?	[45]
98	HuB1-1	x	x	x	x	-	-	-	Environmental isolate	[17]
101	IS075	x	-	-	-	-	-	x	Intestine of bank vole, Poland	[21]
103	LCT-BC244	x	-	-	-	-	-	-	China General Microbiological Culture	[46]
107	MSX-A1	x	x	x	-	-	-	-	Antarctic concordia station and ISS	[3]
108	MSX-A12	x	-	-	-	-	-	-	Antarctic concordia station and ISS	[3]
111	NVH0597-99	x	-	x	-	-	-	-	Spice mix, food poisoning outbreak, Norway	Sequenced by TIGR
114	R3098/03	x		-	x	-	-	-	Septicemia, UK	[2]
120	Rock4-2	x	x	x	x	-	-	-	Soil, Rockville in Maryland	[2]
122	SJ1	x	-	x	-	-	-	-	?	University of Arizona
124	VD022	x	x	-	-	-	-	-	Water, Belgium	[17]
125	VD045	x	x	x	-	-	-	-	Soil, Denmark	[17]
127	VD102	x	-	x	-	-	-	-	Soil, Guadeloupe	[17]
128	VD142	x	x	-	-	-	-	-	Soil, Scotland	[17]

130	VD154	x	x	x	-	-	-	-	Soil, Abu Dhabi, UAE	[17]
132	VD166	x	x	x	-	-	-	-	Dubai, UAE	[17]
136	VDM034	x	x	-	x	-	-	-	Soil, Spain	[17]
138	W	x	x	x	-	-	-	-	?	Sequenced by TIGR
146	AH1272	x	x	-	x	-	-	-	Amniotic fluid, Iceland	[2]
147	AH1273	x	x	-	x	-	-	-	Human blood, Iceland	[2]
151	Bm Rock1-4	x	x	-	x	-	-	-	Soil, Maryland	[2]
153	BAG2X1-1	x	x	-	x	-	-	-	Soil, Massachusetts	[3]
158	BAG6O-2	x	x	-	x	-	-	-	Soil, Massachusetts	[3]
160	BAG6X1-2	x	x	-	-	-	-	-	Soil, Massachusetts	[3]
171	HuA2-1	x	x	-	-	-	-	-	Environmental isolate	[17]
172	HuA4-10	x	x	-	x	-	-	-	Environmental isolate	[17]
179	VD107	x	x	-	-	-	-	-	Soil, Guadeloupe	[17]
188	VD118	x	x	-	x	-	-	-	Soil, Guadeloupe	[17]
196	BAG2X1-3	x	x	-	x	-	-	-	Soil, Massachusetts	[3]
200	BAG1X2-3	x	-	x	x	-	-	-	Soil, Massachusetts	[3]
203	BAG1X1-1	x	-	x	x	-	-	-	Soil, Massachusetts	[3]
204	K-5975c	x	x	-	x	-	-	-	Pasta salad, fatal food poisoning, 2003, Belgium	[47]
207	VD140	x	-	x	-	-	-	-	Soil, Scotland	[17]
216	B5-2	x	x	-	-	-	-	-	Soil, China	[17]
217	BAG1O-1	x	x	-	-	-	-	-	Soil, Massachusetts	[3]
218	BAG3O-1	x	x	-	-	-	-	-	Soil, Massachusetts	[3]
219	BAG5X12-1	x	x	x	-	-	-	-	Soil, Massachusetts	[3]
221	HuA2-9	x	x	-	-	-	-	-	Environmental isolate	[17]
222	HuA3-9	x	-	-	-	-	-	-	Environmental isolate	[17]
223	HuB4-4	x	x	-	-	-	-	-	Environmental isolate	[17]
224	ISP2954	x	-	x	-	-	-	-	Food (durum wheat), Belgium	[17]
226	Schrouff	x	x	-	-	-	-	-	Milk, Belgium	[17]
227	TIAC219	x	x	-	-	-	-	-	Spaghetti and tomato sauce, lethal intoxication,	[48]
231	VD146	x	x	-	-	-	-	-	Soil, Scotland	[17]
233	VDM053	x	x	-	-	-	-	-	Water, Belgium	[17]
234	BAG1O-3	x	-	x	-	-	-	-	Soil, Massachusetts	[3]
290	H3081.97	x	-	-	x	-	-	x	Environmental isolate, USA, CDC	Sequenced by J. Craig Venter Institute

Table S3: Primer used for transcription analysis of *nhe* and nhe_a.

Primer	Target	Sequence [5‘ – 3‘]	Source
16Sf	Inner fragment of 16S rRNA gene *rrn*	GAC GTC AAA TCA TCA TGC C	This work
16Sr		GAT TCC AGC TTC ATG TAG G	
nheAaf	Inner fragment of $nheA_a$	CTA GTA AAG TTA GCA GAG CG	This work
nheAar		TTT CTT TTG GTA GAG CTA GAA G	
nheBaf	Inner fragment of $nheB_a$	TTA TAT TGC ATC GTC GGT TG	This work
nheBar		TTA TCT GCT GCT GCG ATG	
nheCaf	Inner fragment of $nheC_a$	CTA GAT AAC GTG GTG GC	This work
nheCar		TTC CGT TTT ATT TTT GGC ATC	
nheA_qRT_for	Inner fragment of *nheA*	AAG TAC AAA GCA TCC AAG AGA	This work
nheA_qRT_rev		ACA ATA TCT CCA CTT GAT CCT T	
nheB_qRT_for	Inner fragment of *nheB*	GTG AAA CAA GCT CCA GTT C	[49]
nheB_qRT_rev		AAA GCG TAC AGA TCC ATT ACT	
nheC_qRT_for	Inner fragment of *nheC*	GCA AAT GCA GAA A/CGA GAA AT	This work
nheC_qRT_rev		CCT ACT GTA TAC CAT TGA TTT GA	

Table S4: Intra-operon recombination analysis of enterotoxin operons.

142 concatenated *nheABC* genes, 94 concatenated *hblCDAB* genes and 46 concatenated *hblCDA$_a$* genes were investigated. Statistically proven recombination events detected by RDP3 (see Material and Methods). Strains containing two recombinations are highlighted in bold, strains containing three recombinations are additionally underlined. * breakpoint unclear. All breakpoints of recombination events were independent of gene boundaries.

Thirteen *nhe* intra-operon recombinations involved cluster III strains and all strains containing two recombinations are also found in cluster III. In cluster VI a group of closely related strains (#61, #85, #87, #97 and #137) contains three recombinations.

Recombinant sequence(s) *nheABC*	Breakpoints [bp]	Cluster	Minor parent	Major parent	#programs	Max. average p-value
212, **113**, 104, 109, 139, 143, 246	64 – 2256	III & II	140	73	7	1,00E-07
117, 129, 44, 100, 115, 210, 237, 289, 52, 99, 118, 209, 54, 55, 173, 220	1325 – 2202	IV & V	213	180	7	2,30E-02
62, 102	18 – 2371	III & IV	119	16	7	4,56E-03
70, 75, 90, 119, **1**	1113 – 2424	III	84	91	7	9,30E-03
**113**	65* – 515	III	83	246	5	3,51E-04
211, 144, 256	985 – 2446	II	140	56	7	2,89E-04
280, 15, 58, 59, 63, 4, 11, 64, 71, 72, 82, 214, 116	1995 – 3031	IV	199	240	5	7,62E-03
69, 121, 52, 99, 118, 209, 54, 55, 173, 220	87 – 1317*	V & III	117	91	5	1,52E-03
93	38 – 1954	III	148	83	7	1,07E-03
**85, 97, 61, 87, 137** (underlined)	188 – 1294	V & III	177	93	7	1,78E-02
235, 78, 134, 133	1581 – 2212	IV	238	236	4	8,16E04
56, 47	1059 – 2791	II & III	140	242	6	2,29E-03
83	995 – 1582	III	148	206	6	2,59E-02
174	2355 – 3030	VI	155	126	4	2,18E-03
**1, 91**	386 – 819	III	242	5	5	4,56E-02

73	2631 – 2907	III & IV	90	247	4	8,18E-03
141	1996* – 2790	III & IV	12	4	3	1,47E-02
**85, 97, 61, 87, 137**	1295* – 2008	II & IV	47	135	4	2,38E-02
**91**	1686 – 2034	IV & III	241	28	3	2,23E-03
10, 126	1025 – 1327	VI	149	174	7	3,67E-02
**85, 97, 61, 87, 137**	2009* – 2662	VI	177	10	6	4,21E-02

**Recombinant sequence(s) *hblCDAB***	**Breakpoints [bp]**	**Cluster**	**Minor parent**	**Major parent**	**# programs**	**Max. average p-value**
126, 61, 87, 10	2041 – 4892	VI	166	155	7	1,04E-18
174	3681 – 4863*	VI	61	10	7	6,82E-04
229, 69, 54	674 – 1685	V	55	180	4	2,44E-03
280	2867 – 4962	IV	59	241	3	2,58E-03
236, 65, 95, 213	2138 – 3421	IV	116	16	3	3,14E-02

**Recombinant sequence(s) *hblCDA$_a$***	**Breakpoints [bp]**	**Cluster**	**Minor parent**	**Major parent**	**# programs**	**Max. average p-value**
144	1325 – 2475	II & VI	232	140	6	8,27E-04
10, 126, 85, 97	2173 – 2362	II & V	180	140	3	1,47E-02
135, 177, 137, 232, 283, 61, 87, 149, 166, 168	2180 – 2362	II & V	180	140	3	1,47E-02

Presence of second *nhe*$_a$ operon in newly sequenced strains

To confirm the presence of the second *nhe*$_a$ operon in some of the newly sequenced strains trimmed and quality filtered read data was aligned separately against each of the suspicious *nhe* operons as well as the contigs on which the operons are located on. Read alignment was performed using BWA v.0.7.12 [50]. Subsequently, the resulting SAM files were converted into BAM format, whereby reads not mapping to the reference or not being part of a primary alignment to the reference were discarded. SAM file conversion and filtering was carried out using the 'view' utility of the SAMtools package v.0.1.18 [51]. Filtered BAM files served as input for the 'genomeCoverageBed' utility of the BEDTools suite v.2.17.0 [52] to obtain per-base sequencing depths of respective references in BED files. Sequencing depth histograms (BED files) were used to calculate each reference's median coverage.

Table S5 summarizes the median coverage information obtained for each operon (cov_{operon}) and the contig (cov_{contig}) it belongs to. Taking the ratio of cov_{operon} to cov_{contig} shows that all operons fit very well to their genomic backgrounds (contigs), since respective values are close to 1. Significant read pile-up beyond genomic backgrounds of individual operons is not observable, since *nhe*$_a$ diverged from *nhe* to a point that its reads do not align to *nhe* anymore (and vice versa). Examination of read sets mapping to *nhe*$_a$ and *nhe* indeed shows that most reads either map to *nhe*$_a$ or *nhe* (# unique reads) with only a small number of reads mapping to both copies (# combined reads).

Table S5: Confirmation of presence of second nhe_a operons.

Three of the *de novo* assembled strains were found to contain nhe_a, which is discerned from *nhe* by its uniquely mapping reads.

* The *nhe* operon maybe be located within the wrong contig due to an unexpected high ratio ($cov_{operon}/cov_{contig}$) of 1.44. However, examination of read sets mapping uniquely either to *nhe* or nhe_a unambiguously show that both versions are present within the genome of strain #87.

** Unusually high read number is caused by a ~ 40x higher coverage over the intergenic region (2474 – 2634 bp) between *nheB* and *nheC*, which may be due to a duplication of this region into a plasmid with high copy numbers.

Strain	Operon	Median operon coverage (cov_{operon})	Median contig coverage (cov_{contig})	Ratio ($cov_{operon}/cov_{contig}$)	# Unique reads	# Combined reads
#87	*nhe*	155	108	1.44*	2,996	40
	nhe_a	153	144	1.06	3,183	
#144	*nhe*	123	127	0.97	9,893**	4
	nhe_a	235	231	1.02	4,800	
#140	*nhe*	59	62	0.95	950	8
	nhe_a	66	66	1.00	1,106	

Presence of second hbl_a operon in newly sequenced strains

To confirm the presence of the second hbl_a operon in some of the newly sequenced strains the same approach of read remapping and filtering as for the *nhe* operons was applied. In addition to mapping reads to *hbl*, hbl_a and the contigs the operons are located on, reads were also mapped to an artificial sequence construct separating each strain's version of *hbl* and hbl_a by a sequence of 5,000 'N' characters.

Table S6 summarizes the median coverage information obtained for each operon (cov_{operon}) and the contig (cov_{contig}) it belongs to as well as the median coverages of *hbl* and hbl_a within artificial sequence constructs ($cov_{construct}$). Taking the ratio of $cov_{construct}$ to cov_{contig} shows that all operons fit very well to their genomic backgrounds (contigs), since respective values are close to 1. Taking the ratio of cov_{operon} to $cov_{construct}$ for each individual operon shows that median coverages obtained after remapping against operon sequences alone are higher than compared to the ones after remapping against corresponding artificial constructs, since respective ratio values are greater than 1. This can be explained by the fact that within each artificial construct reads are preferentially forming primary alignments (best hits) to the operon (*hbl* or hbl_a) where they naturally are originating from. In contrast, when mapping against individual operon sequences alone (no construct!), a substantial fraction of reads originating from hbl_a are aligning to *hbl* as well, but only due to the missing possibility of forming a better alignment with hbl_a (since it is not present). This observation accounts also in vice versa direction.

The third copy of *hbl* in strain #245 is due to an assembly error. On the one hand, an extremely low ratio ($cov_{construct}/cov_{contig}$) of 0.04 was found. On the other hand, there are almost no reads mapping uniquely to the third *hbl* copy, revealing it as a mis-assembled second copy of hbl_a.

Table S6: Confirmation of presence of second *hbl*$_a$ operons.

Five of the *de novo* assembled strains were found to contain two versions of *hbl*, which could be discerned by ratios of cov_{operon} to $cov_{construct}$ being greater than 1.

* Reads that map uniquely to *hbl*$_a$ contain reads of *hbl*$_a$ and *hblCD*. Therefore, coverage is increased in comparison to cov_{contig}.

** Extremely high coverage over the contig (length 4105 bp) suggests that it might be (part of) a plasmid.

*** In a construct containing *hbl*$_a$ and *hblCD* few reads map uniquely against *hblCD*.

Strain	Operon	Median construct coverage ($cov_{construct}$)	Median contig coverage (cov_{contig})	Ratio ($cov_{construct}/cov_{contig}$)	Median operon coverage (cov_{operon})	Ratio ($cov_{operon}/cov_{construct}$)
#245	*hbl*	51	50	1.02	68	1.33
	hbl$_a$	180	157	1.15	210	1.17
	hbl	58	50	1.16	68	1.17
	hblCD	202	1,587**	0.13	222	1.10
	hbl$_a$	196	157	1.25*	210	1.07
	hblCD	60	1,587**	0.04***	222	3.70***
#243	*hbl*	79	83	0.95	93	1.18
	hbl$_a$	76	74	1.03	126	1.66
#283	*hbl*	103	104	0.99	116	1.13
	hbl$_a$	108	109	0.99	156	1.44
#87	*hbl*	102	108	0.94	132	1.29
	hbl$_a$	95	108	0.88	169	1.78
#144	*hbl*	101	101	1.00	123	1.22
	hbl$_a$	102	101	1.01	157	1.54

References

1. Nakamura KJ, M. A.: **Clarification of the Taxonomy of *Bacillus mycoides***. *International journal of systematic bacteriology* 1995, **45**(1):4.
2. Zwick ME, Joseph SJ, Didelot X, Chen PE, Bishop-Lilly KA, Stewart AC, Willner K, Nolan N, Lentz S, Thomason MK *et al*: **Genomic characterization of the *Bacillus cereus* sensu lato species: backdrop to the evolution of *Bacillus anthracis***. *Genome research* 2012, **22**(8):1512-1524.
3. Van der Auwera GA, Feldgarden M, Kolter R, Mahillon J: **Whole-Genome Sequences of 94 Environmental Isolates of Bacillus cereus Sensu Lato**. *Genome announcements* 2013, **1**(5).
4. Rasko DA, Altherr MR, Han CS, Ravel J: **Genomics of the *Bacillus cereus* group of organisms**. *FEMS microbiology reviews* 2005, **29**(2):303-329.
5. Keim P, Price LB, Klevytska AM, Smith KL, Schupp JM, Okinaka R, Jackson PJ, Hugh-Jones ME: **Multiple-locus variable-number tandem repeat analysis reveals genetic relationships within *Bacillus anthracis***. *Journal of bacteriology* 2000, **182**(10):2928-2936.
6. Chun JH, Hong KJ, Cha SH, Cho MH, Lee KJ, Jeong DH, Yoo CK, Rhie GE: **Complete genome sequence of Bacillus anthracis H9401, an isolate from a Korean patient with anthrax**. *Journal of bacteriology* 2012, **194**(15):4116-4117.
7. Lund T, Granum PE: **Characterisation of a non-haemolytic enterotoxin complex from *Bacillus cereus* isolated after a foodborne outbreak**. *FEMS microbiology letters* 1996, **141**(2-3):151-156.
8. Haggblom MM, Apetroaie C, Andersson MA, Salkinoja-Salonen MS: **Quantitative Analysis of Cereulide, the Emetic Toxin of Bacillus cereus, Produced under Various Conditions**. *Applied and Environmental Microbiology* 2002, **68**(5):2479-2483.
9. Helgason E, Okstad OA, Caugant DA, Johansen HA, Fouet A, Mock M, Hegna I, Kolsto AB: ***Bacillus anthracis, Bacillus cereus,* and *Bacillus thuringiensis* - one species on the basis of genetic evidence**. *Appl Environ Microbiol* 2000, **66**(6):2627-2630.
10. Rasko DA, Ravel J, Okstad OA, Helgason E, Cer RZ, Jiang L, Shores KA, Fouts DE, Tourasse NJ, Angiuoli SV *et al*: **The genome sequence of *Bacillus cereus* ATCC 10987 reveals metabolic adaptations and a large plasmid related to *Bacillus anthracis* pXO1**. *Nucleic Acids Res* 2004, **32**(3):977-988.
11. Klee SR, Brzuszkiewicz EB, Nattermann H, Bruggemann H, Dupke S, Wollherr A, Franz T, Pauli G, Appel B, Liebl W *et al*: **The genome of a Bacillus isolate causing anthrax in chimpanzees combines chromosomal properties of B. cereus with B. anthracis virulence plasmids**. *PloS one* 2010, **5**(7):e10986.
12. Ji F, Zhu Y, Ju S, Zhang R, Yu Z, Sun M: **Promoters of crystal protein genes do not control crystal formation inside exosporium of *Bacillus thuringiensis* ssp. *finitimus* strain YBT-020**. *FEMS microbiology letters* 2009, **300**(1):11-17.

13. Hernandez E, Ramisse F, Ducoureau JP, Cruel T, Cavallo JD: ***Bacillus thuringiensis* subsp. *konkukian* (serotype H34) superinfection: case report and experimental evidence of pathogenicity in immunosuppressed mice**. *Journal of clinical microbiology* 1998, **36**(7):2138-2139.
14. Cachat E, Barker M, Read TD, Priest FG: **A Bacillus thuringiensis strain producing a polyglutamate capsule resembling that of Bacillus anthracis**. *FEMS microbiology letters* 2008, **285**(2):220-226.
15. Challacombe JF, Altherr MR, Xie G, Bhotika SS, Brown N, Bruce D, Campbell CS, Campbell ML, Chen J, Chertkov O *et al*: **The complete genome sequence of Bacillus thuringiensis Al Hakam**. *Journal of bacteriology* 2007, **189**(9):3680-3681.
16. Turnbull PC, Nottingham JF, Ghosh AC: **A severe necrotic enterotoxin produced by certain food, food poisoning and other clinical isolates of *Bacillus cereus***. *British journal of experimental pathology* 1977, **58**(3):273-280.
17. Hu X, Van der Auwera G, Timmery S, Zhu L, Mahillon J: **Distribution, diversity, and potential mobility of extrachromosomal elements related to the *Bacillus anthracis* pXO1 and pXO2 virulence plasmids**. *Applied and Environmental Microbiology* 2009, **75**(10):3016-3028.
18. Agata N, Ohta M, Yokoyama K: **Production of *Bacillus cereus* emetic toxin (cereulide) in various foods**. *International journal of food microbiology* 2002, **73**(1):23-27.
19. Xiong Z, Jiang Y, Qi D, Lu H, Yang F, Yang J, Chen L, Sun L, Xu X, Xue Y *et al*: **Complete genome sequence of the extremophilic Bacillus cereus strain Q1 with industrial applications**. *Journal of bacteriology* 2009, **191**(3):1120-1121.
20. Jessberger N, Dietrich R, Bock S, Didier A, Martlbauer E: ***Bacillus cereus* enterotoxins act as major virulence factors and exhibit distinct cytotoxicity to different human cell lines**. *Toxicon : official journal of the International Society on Toxinology* 2014, **77**:49-57.
21. Swiecicka I, Fiedoruk K, Bednarz G: **The occurrence and properties of *Bacillus thuringiensis* isolated from free-living animals**. *Letters in applied microbiology* 2002, **34**(3):194-198.
22. Guinebretiere MH, Broussolle V, Nguyen-The C: **Enterotoxigenic Profiles of Food-Poisoning and Food-Borne *Bacillus cereus* Strains**. *Journal of clinical microbiology* 2002, **40**(8):3053-3056.
23. Frankland GC, Frankland PF: **Studies on Some New Micro-Organisms Obtained from Air**. *Philosophical Transactions of the Royal Society B: Biological Sciences* 1887, **178**(0):257-287.
24. Duport C, Thomassin S, Bourel G, Schmitt P: **Anaerobiosis and low specific growth rates enhance hemolysin BL production by Bacillus cereus F4430/73**. *Archives of microbiology* 2004, **182**(1):90-95.

25. He J, Shao X, Zheng H, Li M, Wang J, Zhang Q, Li L, Liu Z, Sun M, Wang S *et al*: **Complete genome sequence of Bacillus thuringiensis mutant strain BMB171**. *Journal of bacteriology* 2010, **192**(15):4074-4075.
26. Lereclus D, Arantes O, Chaufaux J, Lecadet M: **Transformation and expression of a cloned delta-endotoxin gene in *Bacillus thuringiensis***. *FEMS microbiology letters* 1989, **51**(1):211-217.
27. He J, Wang J, Yin W, Shao X, Zheng H, Li M, Zhao Y, Sun M, Wang S, Yu Z: **Complete genome sequence of Bacillus thuringiensis subsp. chinensis strain CT-43**. *Journal of bacteriology* 2011, **193**(13):3407-3408.
28. Hoffmaster AR, Novak RT, Marston CK, Gee JE, Helsel L, Pruckler JM, Wilkins PP: **Genetic diversity of clinical isolates of *Bacillus cereus* using multilocus sequence typing**. *BMC microbiology* 2008, **8**:191.
29. Wang A, Pattemore J, Ash G, Williams A, Hane J: **Draft genome sequence of *Bacillus thuringiensis* strain DAR 81934, which exhibits molluscicidal activity**. *Genome announcements* 2013, **1**(2):e0017512.
30. Murawska E, Fiedoruk K, Bideshi DK, Swiecicka I: **Complete genome sequence of *Bacillus thuringiensis* subsp. thuringiensis strain IS5056, an isolate highly toxic to *Trichoplusia ni***. *Genome announcements* 2013, **1**(2):e0010813.
31. Cheng T, Lin P, Jin S, Wu Y, Fu B, Long R, Liu D, Guo Y, Peng L, Xia Q: **Complete Genome Sequence of Bacillus bombysepticus, a Pathogen Leading to Bombyx mori Black Chest Septicemia**. *Genome announcements* 2014, **2**(3).
32. Jimenez G, Urdiain M, Cifuentes A, Lopez-Lopez A, Blanch AR, Tamames J, Kampfer P, Kolsto AB, Ramon D, Martinez JF *et al*: **Description of *Bacillus toyonensis* sp. nov., a novel species of the *Bacillus cereus* group, and pairwise genome comparisons of the species of the group by means of ANI calculations**. *Systematic and applied microbiology* 2013, **36**(6):383-391.
33. Guan P, Ai P, Dai X, Zhang J, Xu L, Zhu J, Li Q, Deng Q, Li S, Wang S *et al*: **Complete genome sequence of Bacillus thuringiensis serovar Sichuansis strain MC28**. *Journal of bacteriology* 2012, **194**(24):6975.
34. Hoa NT, Baccigalupi L, Huxham A, Smertenko A, Van PH, Ammendola S, Ricca E, Cutting SM: **Characterization of *Bacillus* Species Used for Oral Bacteriotherapy and Bacterioprophylaxis of Gastrointestinal Disorders**. *Applied and Environmental Microbiology* 2000, **66**(12):5241-5247.
35. Lechner S, Mayr R, Francis KP, Pruss BM, Kaplan T, Wiessner-Gunkel E, Stewart GS, Scherer S: ***Bacillus weihenstephanensis* sp. nov. is a new psychrotolerant species of the *Bacillus cereus* group**. *International journal of systematic bacteriology* 1998, **48 Pt 4**:1373-1382.

36. Hoton FM, Fornelos N, N'Guessan E, Hu X, Swiecicka I, Dierick K, Jaaskelainen E, Salkinoja-Salonen M, Mahillon J: **Family portrait of *Bacillus cereus* and *Bacillus weihenstephanensis* cereulide-producing strains**. *Environmental microbiology reports* 2009, **1**(3):177-183.
37. Thorsen L, Hansen BM, Nielsen KF, Hendriksen NB, Phipps RK, Budde BB: **Characterization of emetic *Bacillus weihenstephanensis,* a new cereulide-producing bacterium**. *Applied and Environmental Microbiology* 2006, **72**(7):5118-5121.
38. Lund T, De Buyser ML, Granum PE: **A new cytotoxin from *Bacillus cereus* that may cause necrotic enteritis**. *Molecular microbiology* 2000, **38**(2):254-261.
39. Guinebretiere MH, Auger S, Galleron N, Contzen M, De Sarrau B, De Buyser ML, Lamberet G, Fagerlund A, Granum PE, Lereclus D *et al*: ***Bacillus cytotoxicus* sp. nov. is a novel thermotolerant species of the *Bacillus cereus* Group occasionally associated with food poisoning**. *International journal of systematic and evolutionary microbiology* 2013, **63**(Pt 1):31-40.
40. Antwerpen M, Proenca DN, Ruckert C, Licht K, Kalinowski J, Hanczaruk M, Tiemann C, Grass G: **Draft genome sequence of *Bacillus anthracis* BF-1, isolated from Bavarian cattle**. *Journal of bacteriology* 2012, **194**(22):6360-6361.
41. Harrington R, Ondov BD, Radune D, Friss MB, Klubnik J, Diviak L, Hnath J, Cendrowski SR, Blank TE, Karaolis D *et al*: **Genome Sequence of the Attenuated Carbosap Vaccine Strain of *Bacillus anthracis***. *Genome announcements* 2013, **1**(1).
42. Price EP, Seymour ML, Sarovich DS, Latham J, Wolken SR, Mason J, Vincent G, Drees KP, Beckstrom-Sternberg SM, Phillippy AM *et al*: **Molecular epidemiologic investigation of an anthrax outbreak among heroin users, Europe**. *Emerg Infect Dis* 2012, **18**(8):1307-1313.
43. Ruckert C, Licht K, Kalinowski J, Espirito Santo C, Antwerpen M, Hanczaruk M, Reischl U, Holzmann T, Gessner A, Tiemann C *et al*: **Draft genome sequence of *Bacillus anthracis* UR-1, isolated from a German heroin user**. *Journal of bacteriology* 2012, **194**(21):5997-5998.
44. Miller JM, Hair JG, Hebert M, Hebert L, Roberts FJ, Jr., Weyant RS: **Fulminating bacteremia and pneumonia due to *Bacillus cereus***. *Journal of clinical microbiology* 1997, **35**(2):504-507.
45. Lereclus D, Menou G, Lecadet MM: **Isolation of a DNA sequence related to several plasmids from *Bacillus thuringiensis* after a mating involving the *Streptococcus faecalis* plasmid pAM beta 1**. *Molecular & general genetics : MGG* 1983, **191**(2):307-313.
46. Su L, Zhou T, Zhou L, Fang X, Li T, Wang J, Guo Y, Chang D, Wang Y, Li D *et al*: **Draft genome sequence of Bacillus cereus strain LCT-BC244**. *Journal of bacteriology* 2012, **194**(13):3549.

47. Dierick K, Van Coillie E, Swiecicka I, Meyfroidt G, Devlieger H, Meulemans A, Hoedemaekers G, Fourie L, Heyndrickx M, Mahillon J: **Fatal family outbreak of *Bacillus cereus*-associated food poisoning**. *Journal of clinical microbiology* 2005, **43**(8):4277-4279.
48. Naranjo M, Denayer S, Botteldoorn N, Delbrassinne L, Veys J, Waegenaere J, Sirtaine N, Driesen RB, Sipido KR, Mahillon J *et al*: **Sudden death of a young adult associated with Bacillus cereus food poisoning**. *Journal of clinical microbiology* 2011, **49**(12):4379-4381.
49. Jeßberger N, Krey VM, Rademacher C, Böhm M-E, Mohr A-K, Ehling Schulz M, Scherer S, Märtlbauer E: **From genome to toxicity: a combinatory approach highlights the complexity of enterotoxin production in *Bacillus cereus***. *Front Microbiol* 2015, **6**.
50. Li H, Durbin R: **Fast and accurate short read alignment with Burrows-Wheeler transform**. *Bioinformatics* 2009, **25**(14):1754-1760.
51. Li H, Handsaker B, Wysoker A, Fennell T, Ruan J, Homer N, Marth G, Abecasis G, Durbin R, Genome Project Data Processing S: **The Sequence Alignment/Map format and SAMtools**. *Bioinformatics* 2009, **25**(16):2078-2079.
52. Quinlan AR: **BEDTools: The Swiss-Army Tool for Genome Feature Analysis**. *Current protocols in bioinformatics / editoral board, Andreas D Baxevanis [et al]* 2014, **47**:11 12 11-11 12 34.
