# Supplementary material for: Massive horizontal gene transfer, strictly vertical inheritance and ancient duplications differentially shape the evolution of Bacillus cereus enterotoxin operons hbl, cytK and nhe
Source: BMC Evol Biol. 2015 Nov 10;15:246. doi: 10.1186/s12862-015-0529-4 (PMC4641410; doi:10.1186/s12862-015-0529-4)
Supplement: Additional file 2: — Contains Figures S1-S4. (PDF 1084 kb) [file 12862_2015_529_MOESM2_ESM.pdf]

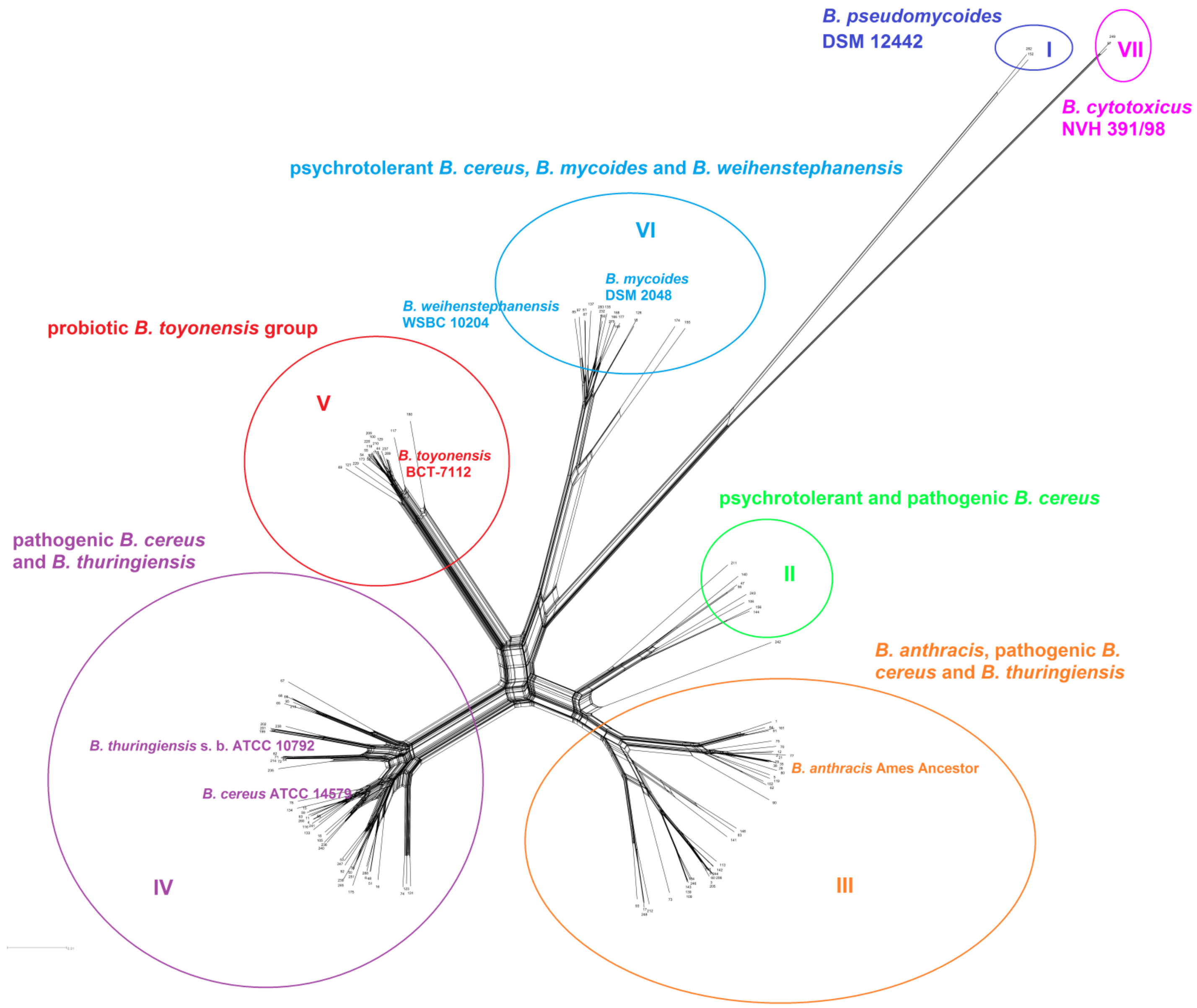
B. pseudomycoides
DSM 12442
I
VII
B. cytotoxicus
NVH 391/98
psychrotolerant B. cereus, B. mycoides and B. weihenstephanensis
VI
B. mycoides
DSM 2048
B. weihenstephanensis
WSBC 10204
probiotic B. toyonensis group
V
B. toyonensis
BCT-7112
psychrotolerant and pathogenic B. cereus
II
pathogenic B. cereus
and B. thuringiensis
B. anthracis, pathogenic B. cereus and B. thuringiensis
B. thuringiensis s. b. ATCC 10792
B. cereus ATCC 14579
B. anthracis Ames Ancestor
IV
III

Fig. S1: ANI-based species relationship within *B. cereus* sensu lato.

Neighbor network was calculated using ANI distances of 142 *B. cereus* sensu lato genomes. Entire genomes (completed or draft) including all available plasmids were used. Phylogenetic groups are designated according to [1]. *B. cereus* F528/94 (#242) is intermediate between clusters II and III.

Ancient duplication of *hbl*

The second *hbl* operon hbl_a is common in clusters II, V and VI and probably was acquired by HGT in cluster IV (Fig. 1, grey crosses; Fig. S2B, grey circles). Several strains seem to have lost their second *hbl* operon and it is not found at all in cluster III. Intra-operon recombination analysis (Table S4) revealed three significant recombination events that all include *B. cereus* MHI 226 (#140, II) as a parental sequence and took place within $hblD_a$. The duplication of *hblCDA* seems to be an ancient and unique event, because it occurs in all phylogenetic groups but III (loss of hbl_a at furcation of II and III) and VII (Fig. 1). The topologies of *hbl* and hbl_a phylogenies are similar but not identical, which could be explained by HGT (Fig. S2 and Fig. 4). Possibly due to directional selection $hblCDA_a$ is as conserved as *hblCDA* (Fig. 4B). Hbl_a shows overall nucleotide sequence identity of 75 – 82 % towards the *hbl* genes, which are 89 – 100 % identical among themselves. Hbl_a are 93 – 100 % identical among each other. Six strains (#85, #97, #137, #140, #152, and #211) possess only *hblCDA*. Their version of *hbl* is homologous to $hblCDA_a$ and they may have lost *hblCDAB*. *B. mycoides* Rock3-17 (#152, I) and *B. mycoides* Rock1-4 possess an *hblCDA* that differs from both *hbl* variants described above. *B. mycoides* Rock3-17 *hblCDA* shows 80 – 82 % identity to $hblCDA_a$, but 86 – 89 % identity to *hblCDA*. We suggest that these two strains have no hbl_a, but their *hblCDAB* developed differently and lost *hblB*.

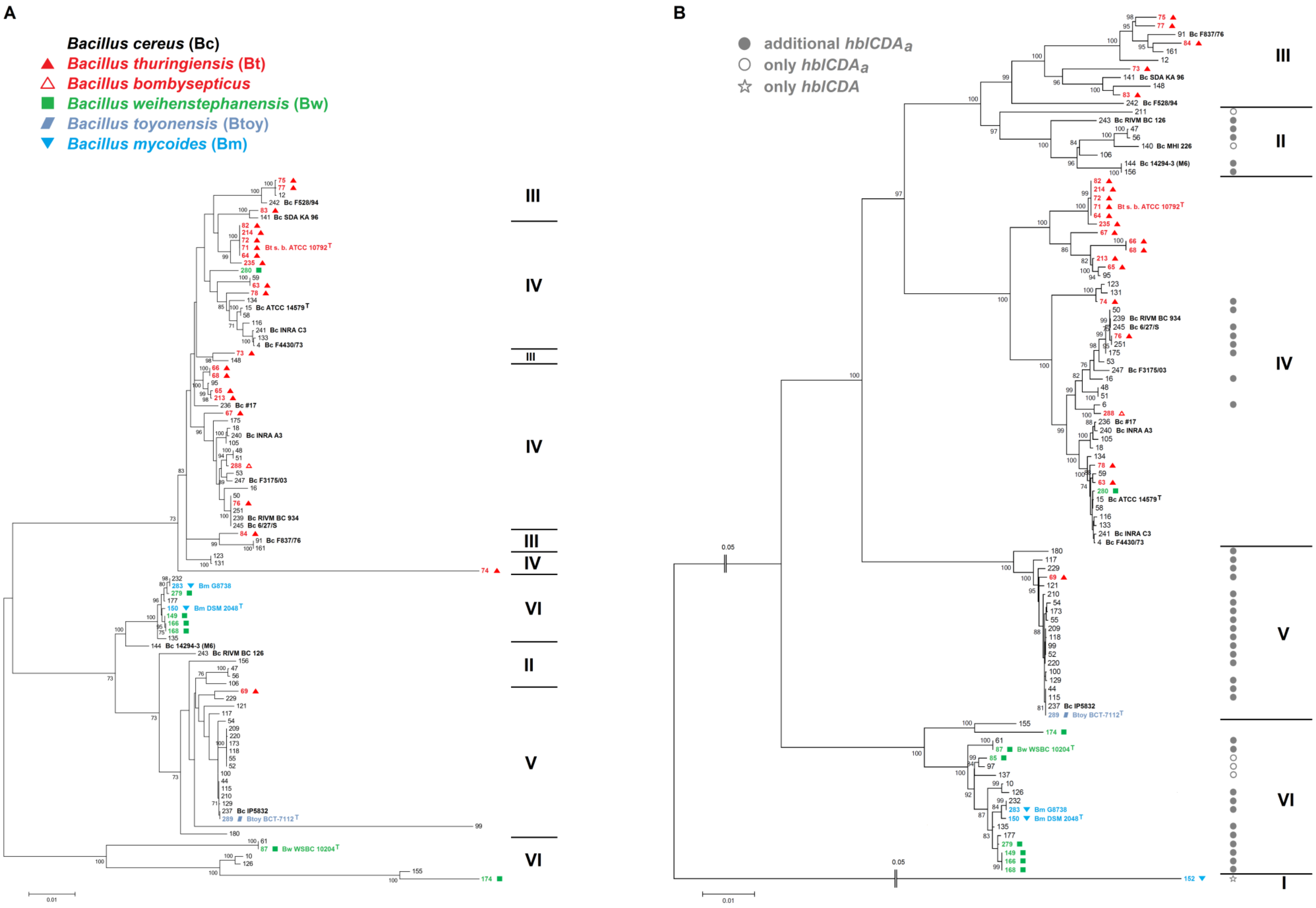

A
Bacillus cereus (Bc)
Bacillus thuringiensis (Bt)
Bacillus bombysepticus
Bacillus weihenstephanensis (Bw)
Bacillus toyonensis (Btoy)
Bacillus mycoides (Bm)
B
additional hblCDA$_a$
only hblCDA$_a$
only hblCDA

Fig. S2: *Hbl* in *B. cereus* sensu lato.

A: Phylogenetic tree (Maximum Likelihood Method) of the concatenated sequences of 94 *hblCDAB* genes. Seven strains within the set 142 contain only *hblCDA* (empty circle and star) or an incomplete *hbl* operon (#6, IV) and were excluded. **B:** Phylogenetic tree based on the seven housekeeping genes of 101 *hbl*-containing *B. cereus* sensu lato strains.

Recombinational mechanism and boundaries of *hbl*

Since *hbl* occurs chromosomally as well as plasmid-bound, we analyzed the immediate vicinity of *hbl* and hbl_a with regard to potential indications of transposon activity in all 16 *hbl*-containing strains that were sequenced in this study. It has been speculated that *hbl* is part of a large 18 kb transposon [2-4]. A comparison of putative transposon regions including *hbl* or hbl_a is shown in Figure S3. Sequence analysis and annotation with RAST [5] revealed that half of the *hblCDAB* operons are inserted within the *uvrC* gene as described earlier [3], but in the rest neither insertion sites nor length of the inserting region or adjacent genes are conserved. The lowest common denominator of inserting regions from 16 *B. cereus* sensu lato strains consists only of a transcriptional regulator gene of the *araC* family and *hblCDAB* itself. Inverted repeats (IR and bcr1) [3, 6], which mark the insertion site interrupting *uvrC* as telltale signs of transposons, could not be found in half of the investigated strains. A transposase gene could only be detected in the vicinity of hbl_a of *B. cereus* 6/27/S (#245, IV), but not adjacent to *hbl* (Fig. S3). Studied hbl_a are located close to antibiotic resistance genes and do not contain known inverted repeats. Thus, one may speculate that the *hbl* operon is part of a highly degraded transposon which is in most cases not functional anymore.

Furthermore, the gene *pagA* encoding a protective antigen similar to a gene located on the *B. anthracis* pXO1 virulence plasmid, has been inserted into the chromosome of *B. cereus, B. mycoides* and *B. weihenstephanensis* (Fig. S3), proving that recombination between virulence plasmids and the bacterial chromosome occurs frequently. *Nhe* and *hbl* duplications occur chromosomally as well as plasmid-bound and, hence, are mobile within *B. cereus* sensu lato.

These results show that recombination within *B. cereus* sensu lato is limited only by preservation of gene/protein functionality. Consequently, the pathogenic potential of (psychrotolerant) environmental strains or probiotic strains can change rapidly with a single and simple exchange of genetic material. This observation may render the current risk assessment strategies questionable.

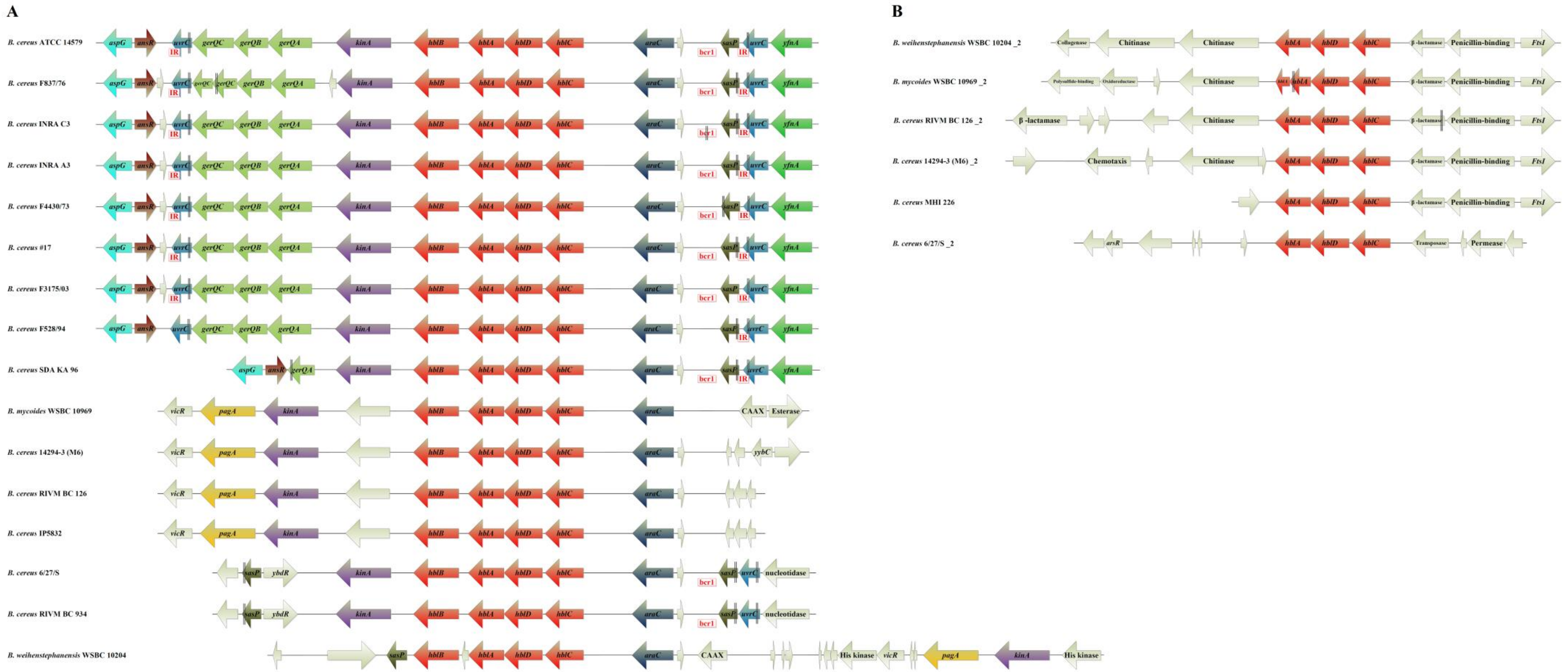

Fig. S3: Genomic organization of *hbl* operons and adjacent regions of 17 *B. cereus* sensu lato strains.

A: 15 of the strains sequenced in this study contain *hblCDAB*, they are shown in comparison to type strain *B. cereus* ATCC 14579.

B: Six of the strains sequenced in this study contain *hblCDA*$_a$.

Gene denomination according to annotation with RAST [5] and previous studies [3, 4, 6]: *ansR*: *ans* (L-aspartase, L-asparaginase, malate utilization/transporter) operon repressor protein, *araC*: AraC family transcriptional regulator TrrA, *arsR*: ArsR family transcriptional regulator, *aspG*: L-asparaginase, β-lactamase: β-

lactamase class A, CAAX: CAAX amino terminal protease family protein, Chemotaxis: Methyl-accepting chemotaxis protein, Esterase: Erythromycin-esterase type I, *ftsI*: cell division protein, *hblCDAB*: hemolysin BL, His kinase: Two component histidine kinase, *gerQABC*: spore germination proteins, *kinA*: sporulation kinase, nucleotidase: 5' Nucleotidase, Oxidoreductase: FAD-dependent pyridine nucleotide disulfide oxidoreductase, *pagA*: protective antigen (anthrax moiety, pXO1), Penicillin-binding: Penicillin-binding protein, Permease: Permease of the drug/metabolite transporter (DMT) family, Polysulfide-binding: Zn-dependent hydroxyacylglutathione hydrolase (polysulfide-binding protein), *sasP*: small acid soluble protein, Transposase: mobile element protein of the IS605 OrfB family transposase, *uvrC*: UvrC-like excinuclease subunit C, *vicR*: DNA-binding response regulator, *ybdR*: zinc-type alcohol dehydrogenase-like protein, *yfnA*: amino acid permease, *yybC*: uncharacterized protein, bcr1: *Bacillus cereus* repeat 1, IR: inverted repeat, ||: incomplete gene, not denominated arrow: hypothetical gene.

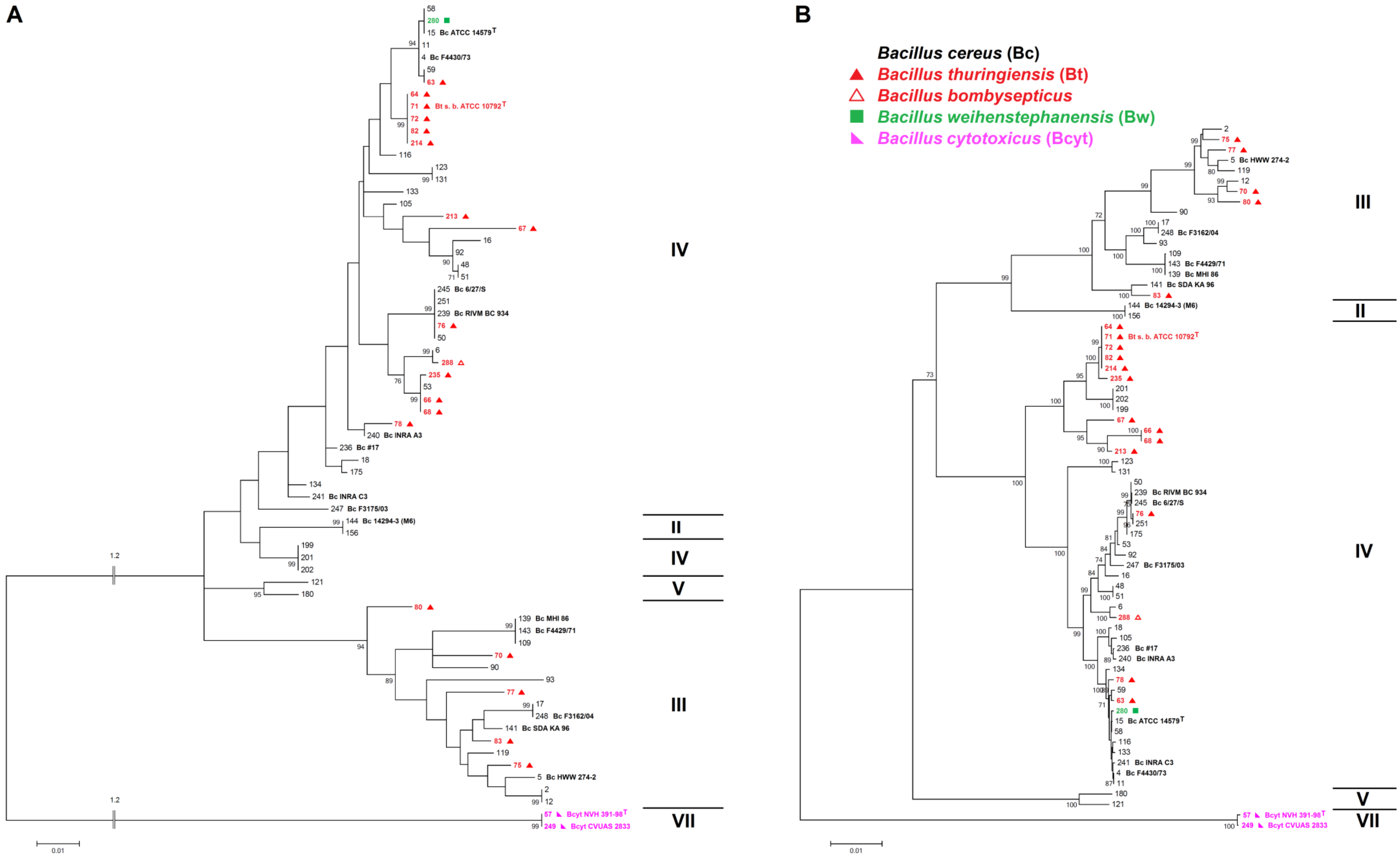
A
B
Bacillus cereus (Bc)
Bacillus thuringiensis (Bt)
Bacillus bombysepticus
Bacillus weihenstephanensis (Bw)
Bacillus cytotoxicus (Bcyt)
Bc ATCC 14579T
Bc F4430/73
Bt s. b. ATCC 10792T
Bc 6/27/S
Bc RIVM BC 934
Bc INRA A3
Bc #17
Bc INRA C3
Bc F3175/03
Bc 14294-3 (M6)
Bc MHI 86
Bc F4429/71
Bc F3162/04
Bc SDA KA 96
Bc HWW 274-2
Bcyt NVH 391-98T
Bcyt CVUAS 2833
II
III
IV
V
VII
0.01

Fig. S4: *CytK* in *B. cereus* sensu lato.

A: Phylogenetic tree (Maximum Likelihood Method) based on 68 *cytK* gene sequences. **B:** Phylogenetic tree based on the concatenated sequence of seven housekeeping genes from 68 *cytK*-containing *B. cereus* sensu lato strains.

References

1. Guinebretiere MH, Thompson FL, Sorokin A, Normand P, Dawyndt P, Ehling-Schulz M, Svensson B, Sanchis V, Nguyen-The C, Heyndrickx M *et al*: **Ecological diversification in the *Bacillus cereus* Group**. *Environmental microbiology* 2008, **10**(4):851-865.
2. Okstad OA, Gominet M, Purnelle B, Rose M, Lereclus D, Kolsto AB: **Sequence analysis of three *Bacillus cereus* loci carrying PIcR-regulated genes encoding degradative enzymes and enterotoxin**. *Microbiology* 1999, **145 (Pt 11)**:3129-3138.
3. Han CS, Xie G, Challacombe JF, Altherr MR, Bhotika SS, Brown N, Bruce D, Campbell CS, Campbell ML, Chen J *et al*: **Pathogenomic sequence analysis of *Bacillus cereus* and *Bacillus thuringiensis* isolates closely related to *Bacillus anthracis***. *Journal of bacteriology* 2006, **188**(9):3382-3390.
4. Stenfors Arnesen LP, Fagerlund A, Granum PE: **From soil to gut: *Bacillus cereus* and its food poisoning toxins**. *FEMS microbiology reviews* 2008, **32**(4):579-606.
5. Aziz RK, Bartels D, Best AA, DeJongh M, Disz T, Edwards RA, Formsma K, Gerdes S, Glass EM, Kubal M *et al*: **The RAST Server: rapid annotations using subsystems technology**. *BMC genomics* 2008, **9**:75.
6. Okstad OA, Hegna I, Lindback T, Rishovd AL, Kolsto AB: **Genome organization is not conserved between *Bacillus cereus* and *Bacillus subtilis***. *Microbiology* 1999, **145 (Pt 3)**:621-631.
